# Shared intentionality modulates interpersonal neural synchronization at the establishment of communication system

**DOI:** 10.1038/s42003-023-05197-z

**Published:** 2023-08-10

**Authors:** Jieqiong Liu, Ruqian Zhang, Enhui Xie, Yixuan Lin, Danni Chen, Yang Liu, Keshuang Li, Mei Chen, Yangzhuo Li, Guanghai Wang, Xianchun Li

**Affiliations:** 1 Shanghai Key Laboratory of Mental Health and Psychological Crisis Intervention, Affiliated Mental Health Center (ECNU), School of Psychology and Cognitive Science, East China Normal University, Shanghai, China; 2 Paediatric Translational Medicine Institute, Department of Developmental and Behavioral Pediatrics, Shanghai Children’s Medical Center, School of Medicine, Shanghai Jiao Tong University, Shanghai, China

**Keywords:** Social neuroscience, Human behaviour

## Abstract

Whether and how shared intentionality (SI) influences the establishment of a novel interpersonal communication system is poorly understood. To investigate this issue, we designed a coordinating symbolic communication game (CSCG) and applied behavioral, functional near-infrared spectroscopy (fNIRS)-based hyperscanning, and hyper-transcranial alternating current stimulation (hyper-tACS) methods. Here we show that SI is a strong contributor to communicative accuracy. Moreover, SI, communicative accuracy, and interpersonal neural synchronization (INS) in the right superior temporal gyrus (rSTG) are higher when dyads successfully establish a novel communication system. Furthermore, the SI influences communicative accuracy by increasing INS. Additionally, using time series and long short-term memory neural network analyses, we find that the INS can predict communicative accuracy at the early formation stage of the communication system. Importantly, the INS partially mediates the relationship between the SI and the communicative accuracy only at the formation stage of the communication system. In contrast, when the communication system is established, SI and INS no longer contribute to communicative accuracy. Finally, the hyper-tACS experiment confirms that INS has a causal effect on communicative accuracy. These findings suggest a behavioral and neural mechanism, subserved by the SI and INS, that underlies the establishment of a novel interpersonal communication system.

## Abstract (web summary)

Shared intentionality is a key factor associated with communicative accuracy and influences the establishment of a non-verbal communication system.

## Introduction

Communication is defined as a process in which people generate meaning through exchanging messages^1^. It plays an essential role in cultivating harmonious interpersonal relationships^2^, improving work efficiency^3^, and maintaining social cohesion^4^. Importantly, individuals with autism spectrum disorder^5,6^ and schizophrenia^7^ are characterized by communication difficulties. However, an outstanding puzzle for researchers is how it arises: How does a novel interpersonal communication system emerge?

In recent decades, scientists have used naturalistic and experimental methods to study how humans create communication systems. Naturalistic studies examine the emergence of human communication by observing home sign communication systems devised by deaf children^8^, language acquisition by infants^9^, and the creation of Nicaraguan Sign Language^10^. However, these naturalistic studies lack experimental control, and it is difficult to identify the critical variables that favor the emergence of human communication. Therefore, experimental researchers attempt to overcome this problem by using the experimental semiotics paradigm to investigate how novel human communication systems might emerge under controlled laboratory conditions. Typically, the experimental semiotics paradigm asks participants to play collaborative games through a symbolic modality, for example, such as drawing^11,12^, gesture^13,14^, or letter-figure mappings^15^. Using these behavioral observation methods, previous studies have identified some factors that influenced the outcome of interpersonal communication. For example, one study examines the mediating role of communication disturbances in the relationship between neuroticism and life satisfaction. It is found that neuroticism significantly affects people’s communication disturbance^16^. Besides, some other researchers have emphasized the crucial role of working memory for semantic information in human verbal communication^17,18^. Additionally, a recent study demonstrates that perspective-taking can improve the accuracy of communication in the success group compared to the failure group during a coordination semiotic game^19^.

In contrast to studies based on traditional behavioral observation methods, neuroscientific methods are also used to study the emergence of a communication system. Stolk et al.^20^ asked each dyad of participants to communicate with each other by moving geometric shapes on a digital board. During the experiment, the neural activity of one participant within each dyad was measured using magnetoencephalography (MEG). Beamforming analysis was conducted to assess neural activity by estimating the time-resolved spectral power of the MEG signals. A key finding is that solving communicative problems elicits comparable neural activity changes in both communicators and addressees. Moreover, this shared neural pattern is spatially localized to the right temporal lobe (TL) and the ventromedial prefrontal cortex (vmPFC)^20^. Furthermore, Stolk et al.^21^ employed the same paradigm but examined the brain activities of two participants simultaneously. They manipulated two types of communicative problems, one is the known condition, and the other is the novel condition. In the known condition, the communicative problems that the participants had to solve were those that they had encountered during the training session prior to the formal experiment. In the novel condition, however, the communicative problems, that participants had to solve, had yet to be previously presented to the dyads. They find that cross-correlation between right superior temporal gyrus (rSTG) activity in real dyads is stronger during episodes involving novel than known problems^21^. These results indicate that converging on conceptual spaces may result in interpersonal neural synchronization (INS) between communicators. Some other studies have consistently demonstrated that INS enhancement between speakers and listeners during verbal communication in some Theory of Mind (ToM) brain regions, including superior temporal gyrus/sulcus (STG/STS), middle temporal gyrus (MTG), A1+, and inferior frontal gyrus (IFG)^22,23^.

From the above review, it can be seen that the creation of a communication system is studied from two aspects, i.e., behavioral science and neuroscience. The studies based on behavioral science show that the communicator’s personality trait^16^, interaction level^13^, perspective taking^19^, and working memory^17,18^ contribute to the communicative outcome. The research based on neuroscience presents that the common neural pattern (i.e., INS) exists in the creation of the communication system^20–23^.

While both of the aforementioned aspects investigate the behavioral factors and neural phenomena associated with the establishment of the communication system, these two types of research are essentially separate. The relationships among the factors (based on behavioral science), the common neural pattern (based on neuroscience), and the effectiveness of communication are not studied. As a result, it cannot find out the mechanism behind the behavioral factors, the shared neural pattern, and the primary motivation factor that contributes to the establishment of a communication system. Moreover, it cannot ensure how a novel interpersonal communication system is established.

According to the “Shared Intentionality Hypothesis,” human communication is a cooperative behavior of human beings, which may rely on shared intentionality or “we” intentionality^24^. Shared intentionality can be described as the cognitive ability to share mental states such as intentions, beliefs, and emotions with others^25^. Several studies have demonstrated that shared intentionality is a key feature of joint action^26^, cooperation^27,28^, and verbal communication^29^ in humans. However, the number of available studies about the impact of shared intentionality on the establishment of a novel communication system is limited, and the knowledge is incomplete.

To address the above issues and to elucidate the mechanism of establishing a communication system, an experimental paradigm called the coordinating symbolic communication game (CSCG) is designed and conducted. Two participants use arbitrary symbols and figures to communicate with each other, share their psychological states, and gradually establish a novel interpersonal symbolic communication system without a pre-established system.

The present study investigates the psychological and neural mechanisms of establishing a novel communication system. To this end, we combined the CSCG with behavioral research (Experiment 1), fNIRS-based hyperscanning technique (Experiment 2), and hyper-tACS stimulation (Experiment 3). First, although the shared intentionality hypothesis mutually assumes that two communicators already have a pre-established shared system^24,29^, it still drives us to hypothesize that high SI also plays a crucial role in the process of establishing a novel communication system. Second, previous studies suggest that INS increases when completing a puzzle together (with SI) as opposed to a condition in which subjects complete identical but individual puzzles (without SI)^30^. In addition, previous studies have shown that INS enhancement in the ToM regions contributes to successful communication^20–23^. Thus, SI will enhance INS in the ToM regions, which will ultimately increase communicative accuracy in the process of establishing a novel communication system. Third, based on the fNIRS results consistent with the above predictions, Experiment 3 further investigated the causal relationship between INS and communicative accuracy by introducing hyper-tACS stimulation. Prior to the CSCG, two participants in a dyad randomly received in-phase, anti-phase, or sham stimulation; we would expect to see this effect to improve INS and communicative accuracy during the coordination period (COP) under the in-phase stimulation compared with anti-phase stimulation and sham stimulation.

Taken together, our behavioral and brain imaging results showed that the SI modulates the INS, which contributes to the establishment of a novel communication system. Furthermore, the SI and INS contribute differently to the communication outcome due to the different stages of establishing the communication system. When the communication system is established, the SI and INS contribute to communication effectiveness. However, when the communication system is already established, SI and INS no longer contribute to communication effectiveness. Finally, in-phase, anti-phase, and sham simulations are carried out to verify that the INS causally contributes to communication effectiveness.

## Results

Experiment 1: shared intentionality is the key psychological process for creating a novel interpersonal communication system

**Fig. 1:**
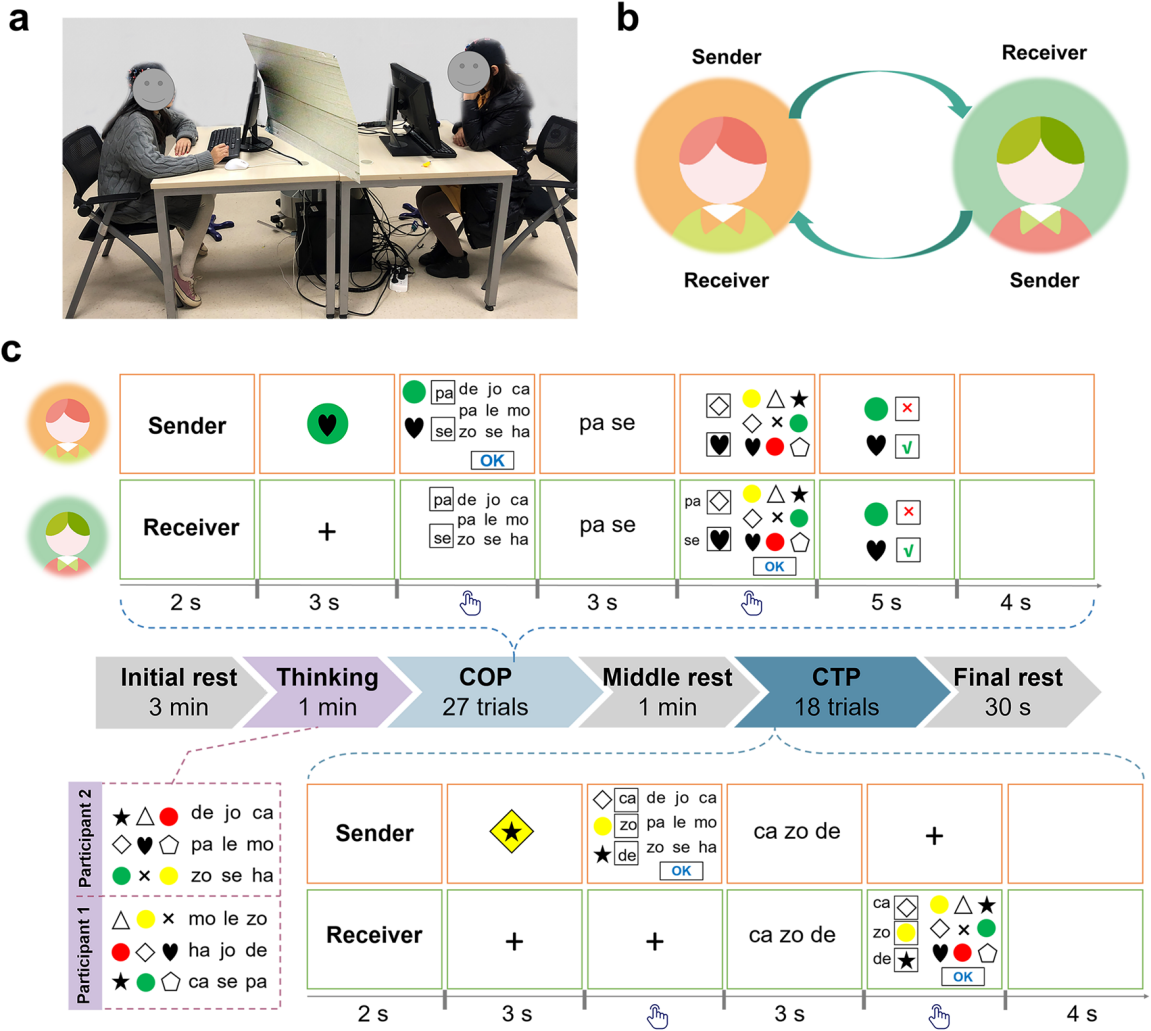
Experiment 1 setup and procedures. **a** Two communicators sat behind separate computer screens across the table, each equipped with a mouse and a keyboard, respectively. Each dyad could not see the other during the task. **b** Two participants alternated as sender or receiver. **c** Timeline for total task periods and task trial sequence for the COP and CTP. The yellow and green square boxes show what the sender and receiver saw, respectively. COP coordination period, CTP communication testing period.

In Experiment 1, a total of thirty participant dyads participated in the CSCG. Two participants sat face-to-face, with two computer screens placed between them and separated by a baffle (Fig. 1a). During the task, participants alternated between the roles of sender and receiver (Fig. 1b). Details of the CSCG procedure can be found in Fig. 1c.

Correlations between communicative accuracy and the Big Five personality traits, working memory span, SI, cooperation, perceived similarity, interpersonal reactivity index, and need for cognition. The SI (*r* = 0.67, *p* < 0.001), cooperation (*r* = 0.41, *p* = 0.026), and perceived similarity (*r* = 0.40, *p* = 0.027) between two communicators were significantly positively correlated with communicative accuracy during COP (Table 1).

**Table 1:** Correlations among variables.

Variables	ACC	1	2	3	4	5	6	7	8	9	10	11	12	13	14
Neuroticism	0.17	–													
Extraversion	−0.27	−0.47**	–												
Openness	−0.20	0.19	0.09	–											
Agreeableness	−0.10	−0.10	0.22	−0.09	–										
Conscientiousness	−0.11	−0.50**	0.40*	0.08	0.01	–									
Shared intentionality	0.67***	0.27	−0.40*	−0.06	0.04	−0.12	–								
Cooperation	0.41*	0.07	0.01	−0.01	−0.10	0.08	0.48*	–							
Similarity	0.40*	0.13	0.07	−0.15	−0.08	0.00	0.40*	0.82***	–						
Perspective-taking	0.24	0.08	0.08	0.14	−0.17	0.31	0.28	0.18	0.11	–					
Fantasizing	0.10	−0.13	−0.11	−0.04	0.04	0.18	0.21	−0.17	−0.29	0.39*	–				
Empathic concern	0.17	−0.07	−0.09	0.01	−0.25	0.28	0.15	0.11	0.02	0.37*	0.63***	–			
Personal distress	0.17	−0.02	−0.10	−0.10	−0.14	−0.08	0.04	−0.19	−0.14	0.00	0.14	0.21	–		
Need for cognition	0.10	−0.07	0.10	−0.21	0.20	0.15	−0.02	0.42*	0.41*	0.10	−0.05	−0.20	−0.45*	–	
Working memory	−0.13	0.08	−0.02	−0.23	0.15	−0.16	−0.10	−0.09	−0.04	−0.14	−0.23	−0.26	−0.04	−0.06	–

*p < 0.05; **p < 0.011; ***p < 0.001.

We performed an automated stepwise linear regression using all statistically significant univariate variables, which is with standard inclusion criteria in every step (*p* of *F* for inclusion ≤0.05; *p* of *F* for exclusion ≥0.1). It demonstrated that shared intentionality (standardized *β* = 0.67, *p* ≤ 0.001) was significantly associated with communicative accuracy during the COP (Table 2). The model implies that among these variables, shared intentionality was the only significant predictor of communicative accuracy during the COP, which could explain 43.5% of the variance in communicative accuracy during the COP (Table 2). These results indicate a key predictive role of shared intentionality in creating a novel interpersonal communication system.

**Table 2:** Results of stepwise regression analysis of factors predicting communicative accuracy.

Independent variables	*B*	SE	*β*	*t*	*p*	Changed in *R*^2^
Shared intentionality	0.027	0.005	0.674	4.832	<0.001	0.435

Experiment 2: INS partially mediates relationships between shared intentionality and communicative accuracy during the COP

**Fig. 2:**
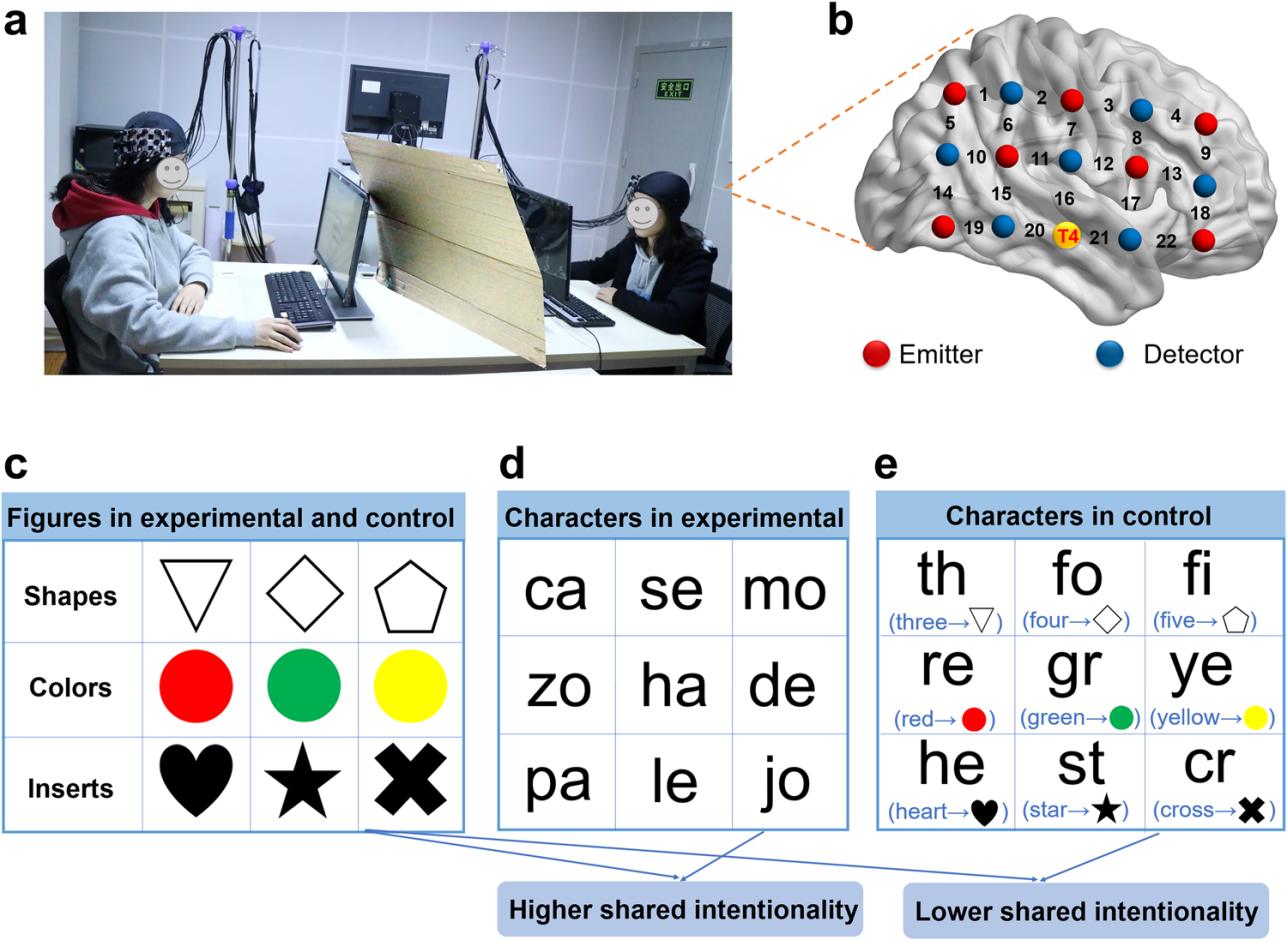
Experiment 2 setup and procedures. **a** The fNIRS hyperscanning environment. The brain activities of two communicators in one dyad were recorded simultaneously using fNIRS. **b** The optode probes were placed on the right temporal-parietal area. T4 (orange circles) in the international 10-20 system was used as reference sites. **c** Figures used in the experimental and control conditions. **d** Characters used under the experimental condition. **e** Characters used under the control condition.

In Experiment 1, shared intentionality was tested after the CSCG, which may be affected by the final experimental results. In addition, we did not explore the differences in shared intentionality and changes in neural processes between the success and failure groups. In Experiment 2, we assessed SI and simultaneously measured the brain activities (Fig. 2a, b) of the senders and receivers from 46 dyads. To manipulate the level of shared intentionality between the dyads, we used different experimental settings. Specifically, we implemented the experimental condition (Higher SI, Fig. 2c, d) and the control condition (Lower SI, Fig. 2c, e). Furthermore, we divided the 43 dyads (3 were removed due to bad signal quality) into the success group and the failure group. The conditions of Experiment 2 and the setup of the optode probes are described in detail in “Stimuli and procedure”. A dyad was assigned to the success group only if two communicators had the exactly same figure-character mappings (*N*_success_ = 21, *N*_failure_ = 22).

### Behavioral results

**Fig. 3:**
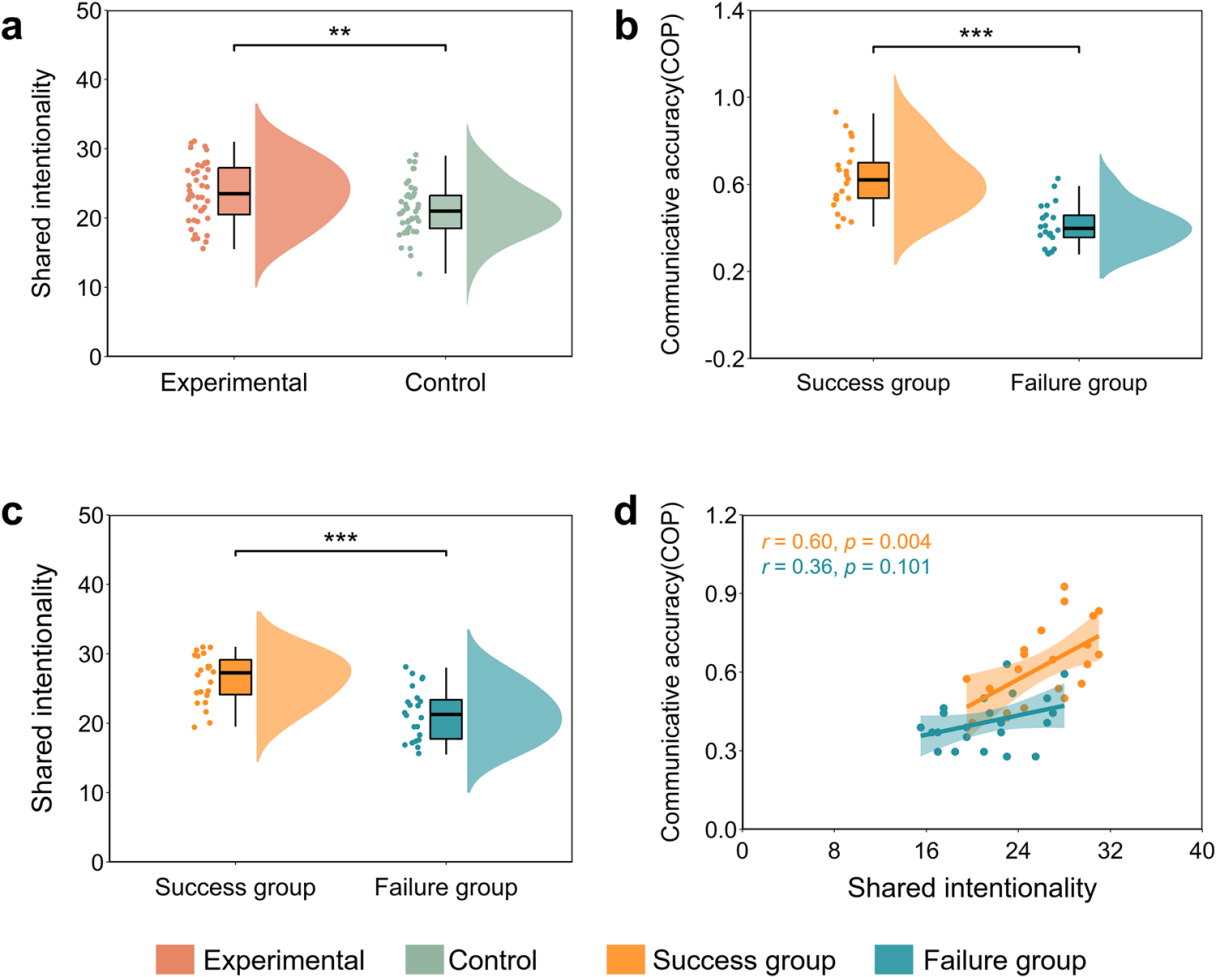
Behavioral results. **a** SI score difference between the experimental condition and the control conditions. **b** Communicative accuracy during the COP difference between the success and failure groups under the experimental condition. **c** SI score difference between the success group and the failure group under the experimental condition. **d** Pearson’s correlation analyses between SI and communicative accuracy during the COP under the experimental condition. Data are plotted as violin and box plots for each group, with white dots indicating median values, boxes indicating 25% and 75% quartiles, and whiskers indicating the 2.5–97.5% percentile range. SI shared intentionality, COP coordination period. **p < 0.01, ***p < 0.001.

To validate the experimental manipulations, the SI of the experimental and the control conditions were compared using a paired samples *t*-test. The experimental condition (*M* ± SE, 23.70 ± 0.66) showed significantly higher levels of SI than the control condition (*M* ± SE, 21.12 ± 0.57), *t*(42) = 3.46, *p* = 0.001, Cohen’s *d* = 0.58 (Fig. 3a). In Experiment 2, we are more interested in the differences between the success group and the failure group during the process of establishing a novel communication system (experimental condition). To identify the communication outcomes, we conducted the independent-samples *t*-test on communicative accuracy during the COP. The results indicated a significant group difference (*t*(41) = 5.73, *p* < 0.001, Cohen’s *d* = 1.74), indicating that the communicative accuracy of the success group (*M* ± SE, 0.63 ± 0.03) was higher than that of the failure group (*M* ± SE, 0.41 ± 0.02; Fig. 3b). Shared intentionality was also compared between the success and failure groups. The independent-samples *t*-test revealed greater shared intentionality in the success group (*M* ± SE, 26.14 ± 0.77) than that in the failure group (*M* ± SE, 21.36 ± 0.81), *t*(41) = 4.29, *p* ≤ 0.001, Cohen’s *d* = 1.31 (Fig. 3c). Furthermore, Pearson’s correlation analysis showed a significant positive correlation between shared intentionality and communicative accuracy during the COP in the success group (*r* = 0.60, *p* = 0.004) but not in the failure group (*r* = 0.36, *p* = 0.101; Fig. 3d). However, Silver’s *z* analysis^31^ showed that these two correlations were not significantly different between the two groups, *z* = 0.97, *p* = 0.332.

### Task related INS in different conditions and groups

**Fig. 4:**
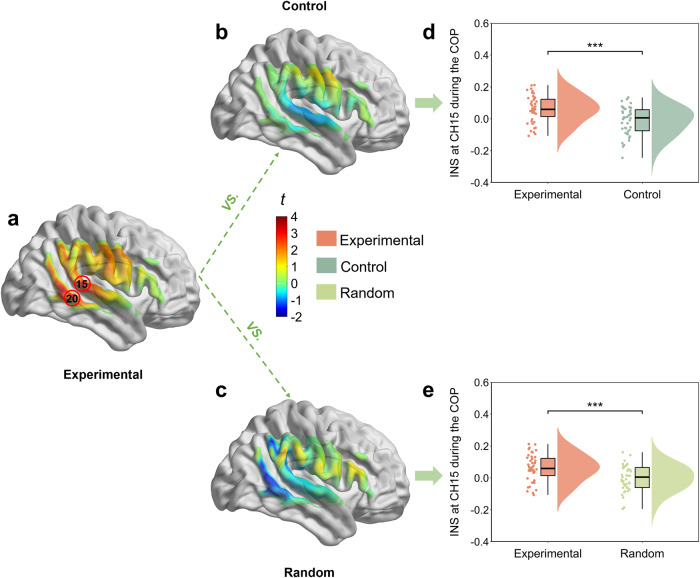
INS enhancement during the task. **a** One-sample *t*-test map of INS in the right temporal-parietal areas under the experimental condition (two-tailed, corrected by FDR). **b** One-sample *t*-test map of INS in the right temporal-parietal regions under the control condition (two-tailed, FDR correction). **c** One sample *t*-test map of INS for the permutated time series based on the original data (two-tailed, FDR correction). **d** The paired samples *t*-test INS at CH15 under different conditions (experiment vs. control). **e** The paired samples *t*-test INS at CH15 under different conditions (experiment vs. random). Data are plotted as violin and box plots for each group, with white dots indicating median values, boxes indicating 25% and 75% quartiles, and whiskers indicating the 2.5–97.5% percentile range. INS interpersonal neural synchronization, COP coordination period. ***p < 0.001.

It should be noted that the control condition was applied with two main goals in the current study. On the one hand, we wondered whether INS enhancement would be found only when a novel symbolic communication system emerged but not when two participants communicated using the existing figure-character system. On the other hand, we also wanted to verify that the INS was not partially increased because participants were exposed to the same stimuli and experimental environment. Indeed, we found that the INS during the COP was significantly higher than baseline in the frequency band ranging from 0.09 to 0.14 Hz (i.e., period 7.04–11.10 s) only under the experimental condition (Supplementary Fig. 1a), but not in the control condition (Supplementary Fig. 1b). Within this FOI, the one-sample *t*-test revealed that INS was significantly enhanced at CH15 (*t*(42) = 4.76, *p* < 0.001, FDR correction; right Superior Temporal Gyrus, rSTG) and CH20 (*t*(42) = 3.82, *p* = 0.005, FDR correction; right Middle Temporal Gyrus, rMTG), under the experimental condition (Fig. 4a), but no significant channel was found in the control condition (one-sample *t*-test, *ps* ≥ 0.05, FDR correction; Fig. 4b).

Furthermore, the paired samples *t*-test was performed on the INS at CH15 during the COP with the condition (experimental vs. control). We found a significant group difference (*t*(42) = 4.17, *p* < 0.001, Cohen’s *d* = 0.87), which indicated that the INS at CH15 under the experimental condition (*M* ± SE, 0.06 ± 0.01) was significantly higher than that in control condition (*M* ± SE, −0.01 ± 0.01; Fig. 4d). Besides, we conducted the same analysis for the INS at CH20 during the COP and found that the experimental condition (*M* ± SE, 0.04 ± 0.01) displayed a significantly stronger INS than the control condition (*M* ± SE, −0.01 ± 0.01), *t*(42) = 2.78, *p* = 0.008, Cohen’s *d* = 0.66 (Supplementary Fig. 2a).

To further verify that the INS enhancement was not obtained by chance, we permutated a time series of each communicator for each dyad. We then reanalyzed the INS on the obtained randomized time series (random condition). The one-sample *t*-test revealed no significant INS enhancement under the random condition (*ts* < 0.85, *ps* > 0.960; Fig. 4c). Then, the paired samples *t*-test was also performed on the INS of CH15 with the condition (experimental vs. random). We observed a significantly higher INS at CH15 under the experimental condition (*M* ± SE, 0.06 ± 0.01) than that in random condition (*M* ± SE, 0.01 ± 0.01), *t*(42) = 2.72, *p* = 0.010, Cohen’s *d* = 0.61 (Fig. 4e). Additionally, we also found greater INS at CH20 during the COP under the experimental condition (*M* ± SE, 0.04 ± 0.01) than that in the random condition (*M* ± SE, 0.01 ± 0.01), *t*(42) = 2.37, *p* = 0.023, Cohen’s *d* = 0.46 (Supplementary Fig. 2b).

**Fig. 5:**
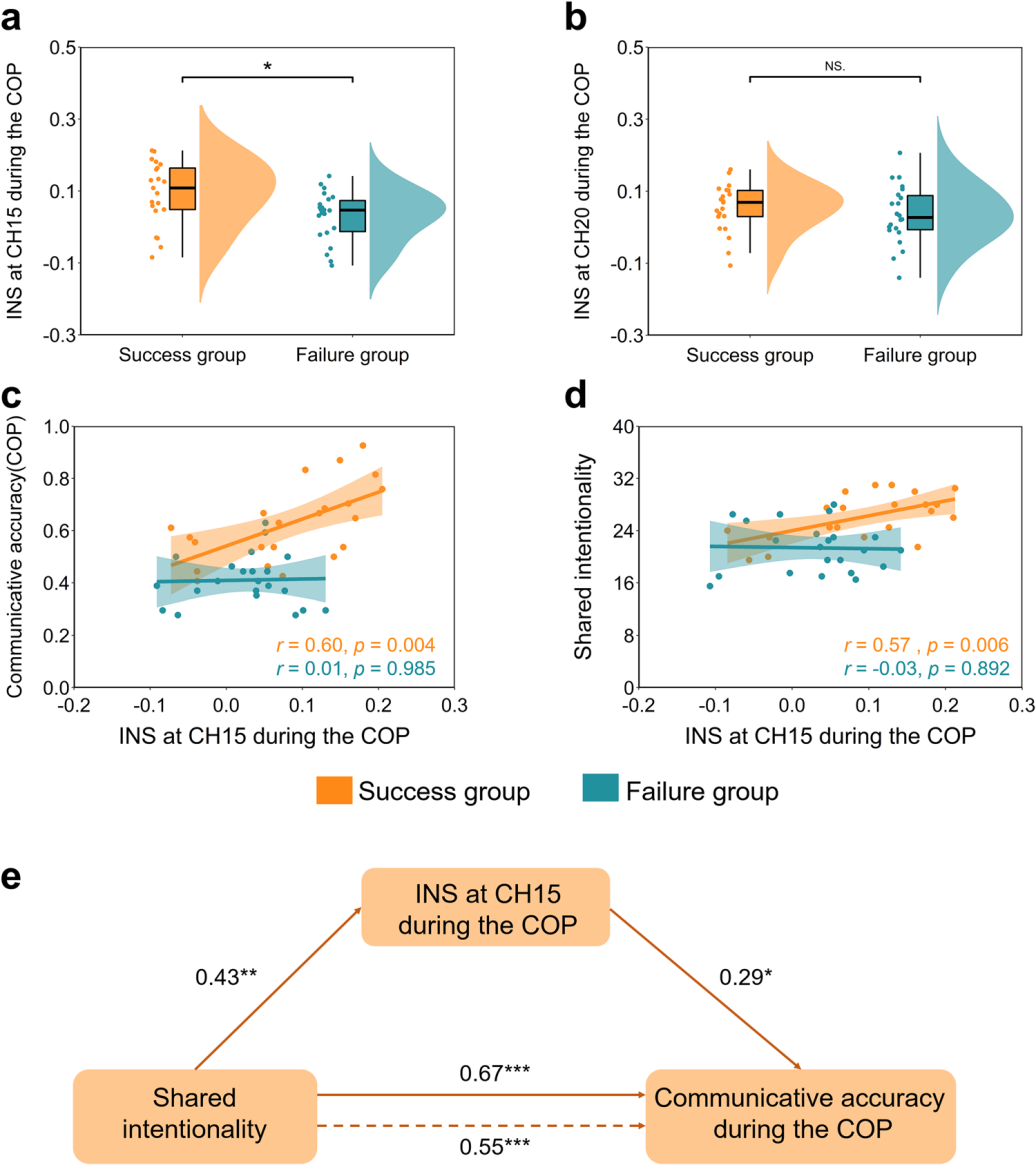
INS enhancement during the task and correlations between behavioral indicators and the INS under the experimental condition. **a** INS at CH15 during the COP difference between the success and failure groups under the experimental condition. **b** INS at CH20 during the COP difference between the success and failure groups under the experimental condition. **c** Pearson’s correlation between INS at CH15 and communicative accuracy during the COP in different groups (success vs. failure) under the experimental condition. **d** Pearson’s correlation between INS at CH15 and SI in different groups (success vs. failure) under the experimental condition. **e** The INS at CH15 mediated the effect of SI on communicative accuracy during the COP under the experimental condition. Data are plotted as violin and box plots for each group, with white dots indicating median values, boxes indicating 25% and 75% quartiles, and whiskers indicating the 2.5–97.5% percentile range. SI shared intentionality, INS interpersonal neural synchronization, COP coordination period. *p < 0.05, **p < 0.01, ***p < 0.001 and NS not significant.

We also conducted the independent-samples *t*-test on communicative accuracy during the COP. The results indicated a significant group difference (*t*(41) = 2.65, *p* = 0.012, Cohen’s *d* = 0.81), indicating that INS at CH15 during the COP in the success group (*M* ± SE, 0.09 ± 0.02) was higher than that in the failure group (*M* ± SE, 0.03 ± 0.01; Fig. 5a) under the experimental condition. Besides, the INS at CH20 during the COP was also compared between the success and failure groups using the same analysis. However, no significant difference was found between the success group (*M* ± SE, 0.05 ± 0.01) and the failure group (*M* ± SE, 0.03 ± 0.02), *t*(41) = 0.80, *p* = 0.426, Cohen’s *d* = 0.24 (Fig. 5b).

Pearson’s correlation analysis showed that the INS enhancement at CH15 during the COP was significantly correlated with communicative accuracy in the success group (*r* = 0.60, *p* = 0.004), but not in the failure group (*r* = 0.01, *p* = 0.985) under the experimental condition (Fig. 5c). Silver’s *z* test^31^ also revealed a significant difference between these two correlations (success group vs. failure group), *z* = 2.07, *p* = 0.039. Moreover, we also observed a positive correlation between the INS at CH15 and shared intentionality in the success group (*r* = 0.57, *p* = 0.006), but not in the failure group (*r* = −0.03, *p* = 0.892; Fig. 5d). The difference between the correlations in the success group and the failure groups was significant (*z* = 2.08, *p* = 0.038). However, the INS enhancement at CH20 during COP was not significantly correlated with either communicative accuracy (Supplementary Fig. 3a) or shared intentionality in either the success group or the failure group under the experimental condition (*rs* < 0.08, *ps* > 0.05; Supplementary Fig. 3b).

### INS partially mediates the effect of shared intentionality on communicative accuracy

Based on these findings, it was plausible to assume a mediating role of increased INS in the relationship between SI and communicative accuracy. To investigate this hypothesis, a mediation analysis was conducted. Figure 5e shows the total effect of shared intentionality on communicative accuracy during the COP (total effect = 0.67, 95% CI = [0.017, 0.035]). And increased INS at CH15 during the COP was positively associated with shared intentionality (*β* = 0.43, *p* = 0.004). Moreover, after controlling for the effect of INS, shared intentionality was still a significant predictor for communicative accuracy (*β* = 0.55, *p* ≤ 0.001), suggesting that the effect of increased INS acted as a partial mediator of the shared intentionality on communicative accuracy (*ab* = 0.13, 95% CI = [0.001, 0.011], obtained from the bootstrapping test; Fig. 5e).

### The dynamic changes of time-cumulative INS and its correlation with the trial-cumulative accuracy

**Fig. 6:**
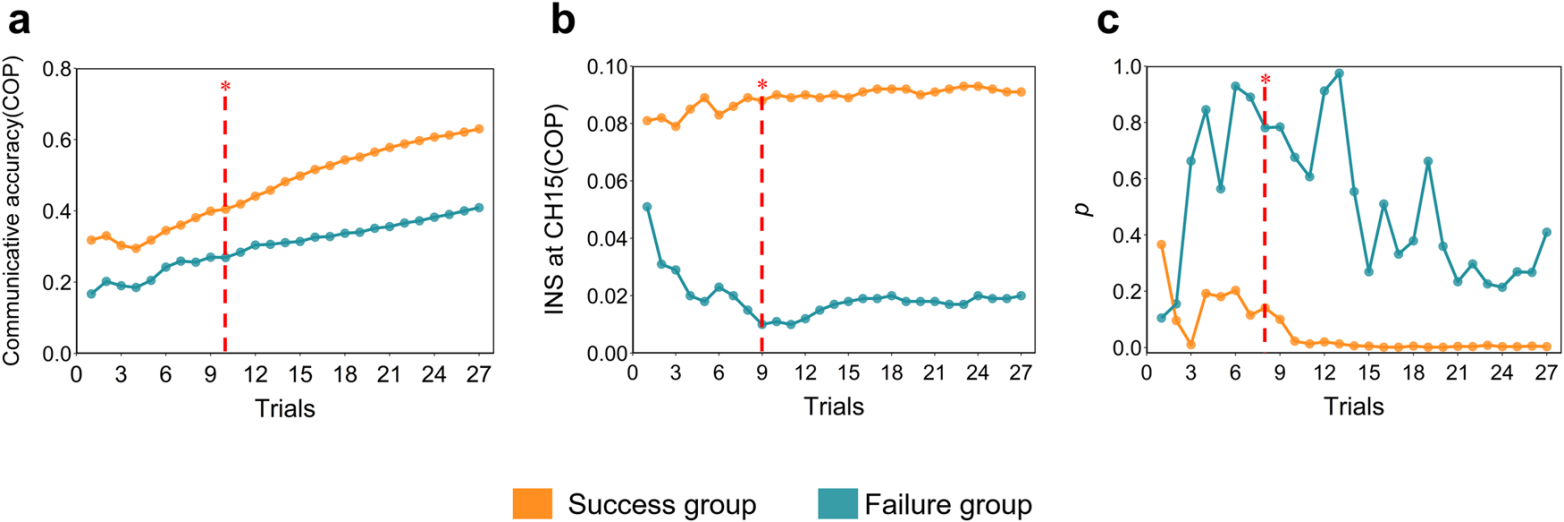
The dynamics of the time-cumulative INS, trial-cumulative accuracy, and correlation results. **a** The trial-cumulative accuracy during the COP in the success and failure groups under the experimental condition. **b** The time-cumulative INS at CH15 during COP in the success and failure groups under the experimental condition. Notably, the black vertical line with an asterisk in A and B indicates the earliest trial in which a significant difference between these two groups was found separately (Wilcoxon signed rank test, *p* < 0.05, FDR correction). **c** Dynamic correlations between the time-cumulative INS and trial-cumulative accuracy during the COP in the success and failure groups under the experimental condition. The red vertical line with one asterisk indicates the earliest trial that the correlations reached statistical significance in the success group (Spearman’s rank correlation, *p* < 0.05, FDR correction). INS interpersonal neural synchronization, COP coordination period. *p < 0.05.

To investigate the earliest trial whose INS enhancement at CH15 differentiated between the success and the failure groups and correlated with communicative accuracy, we conducted the Wilcoxon signed rank test on trial-cumulative accuracy and time-cumulative INS along the trial between the success and failure groups under the experimental condition. For the trial-cumulative accuracy, the trial-cumulative accuracy of the success group was higher than that of the failure group after the 10th trial (*ps* < 0.05, FDR correction; Fig. 6a). Meanwhile, the results demonstrated that the time-cumulative INS during the COP increased in the success group after the 9th trial, as compared with the failure group, under the experimental condition (*ps* < 0.05, FDR correction; Fig. 6b). Furthermore, Spearman’s rank correlation results showed that the time-cumulative INS was significantly correlated with trial-cumulative accuracy after the 8th trial in the success group under the experimental condition (*rs* > 0.52, *ps* < 0.021, FDR correction), but not in the failure group (Fig. 6c).

**Fig. 7:**
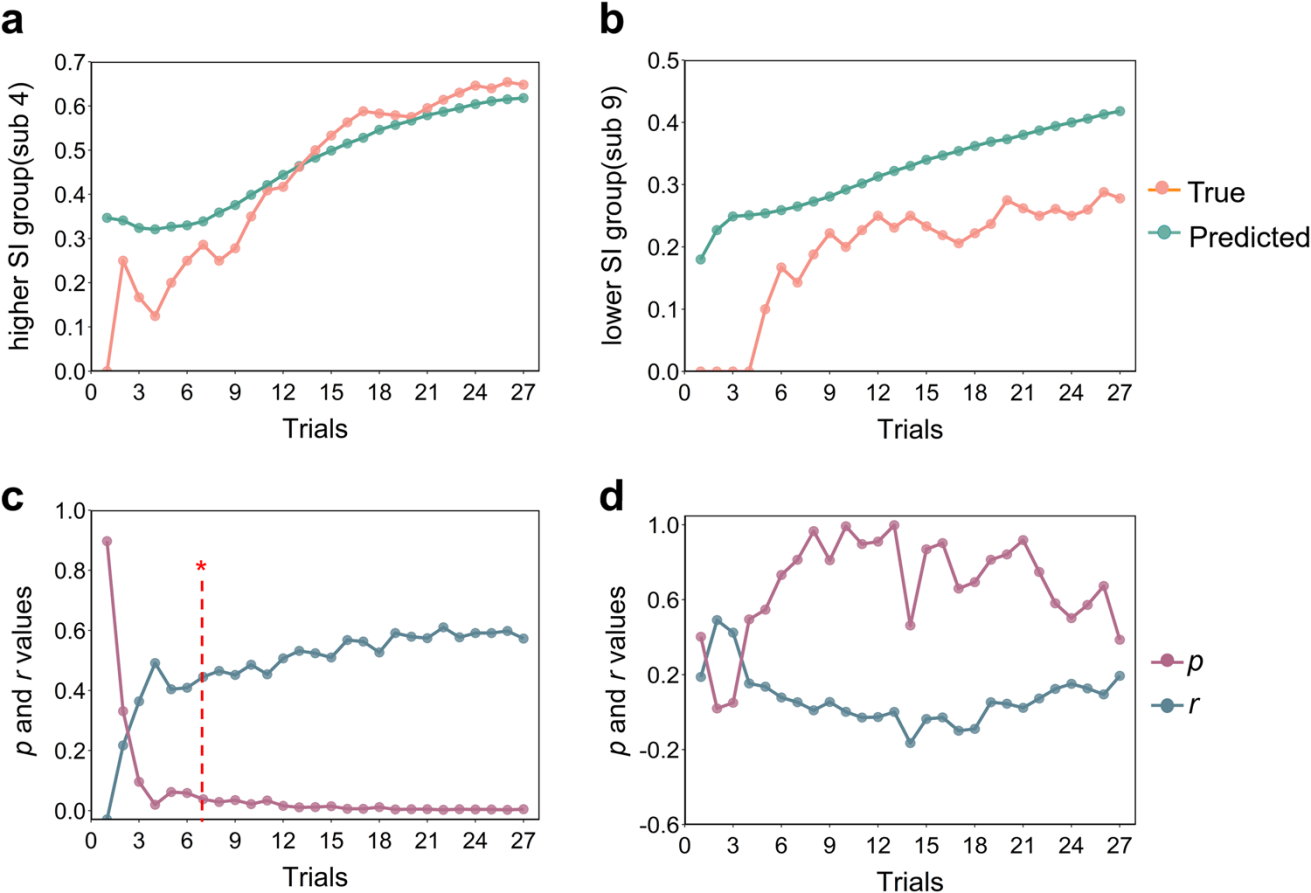
Summary of the LSTM neural network performance. **a** Predicted trial-communicative accuracy and observed trial-communicative accuracy values are shown from a dyad (sub 4) in the success group. **b** Predicted trial-communicative accuracy and observed trial-communicative accuracy values are shown for a dyad (sub 9) in the failure group. **c** Spearman’s rank correlation between predicted trial-communicative accuracy and observed trial-communicative accuracy in the testing dataset in the success group. **d** Spearman’s rank correlation between predicted trial-communicative accuracy and observed trial-communicative accuracy in the testing dataset in the failure group. The red vertical line with an asterisk indicates the earliest trial on which the correlations reached statistical significance in the success group (spearman’s rank correlation, *p* < 0.05, FDR correction).

To examine whether the correlation between time-cumulative INS and trial-cumulative accuracy could predict new dyads, the LSTM neural network was applied in this study. It was found that the INS of CH15 could well predict the communicative accuracy during the COP from the 7th trial in the success group (Spearman’s rank correlation, *rs* > 0.41, *ps* < 0.05; Fig. 7a, c), but not in the failure group (Spearman’s rank correlation, *ps* > 0.05; Fig. 7b, d).

Experiment 3: Manipulating of INS by transcranial alternating current stimulation

**Fig. 8:**
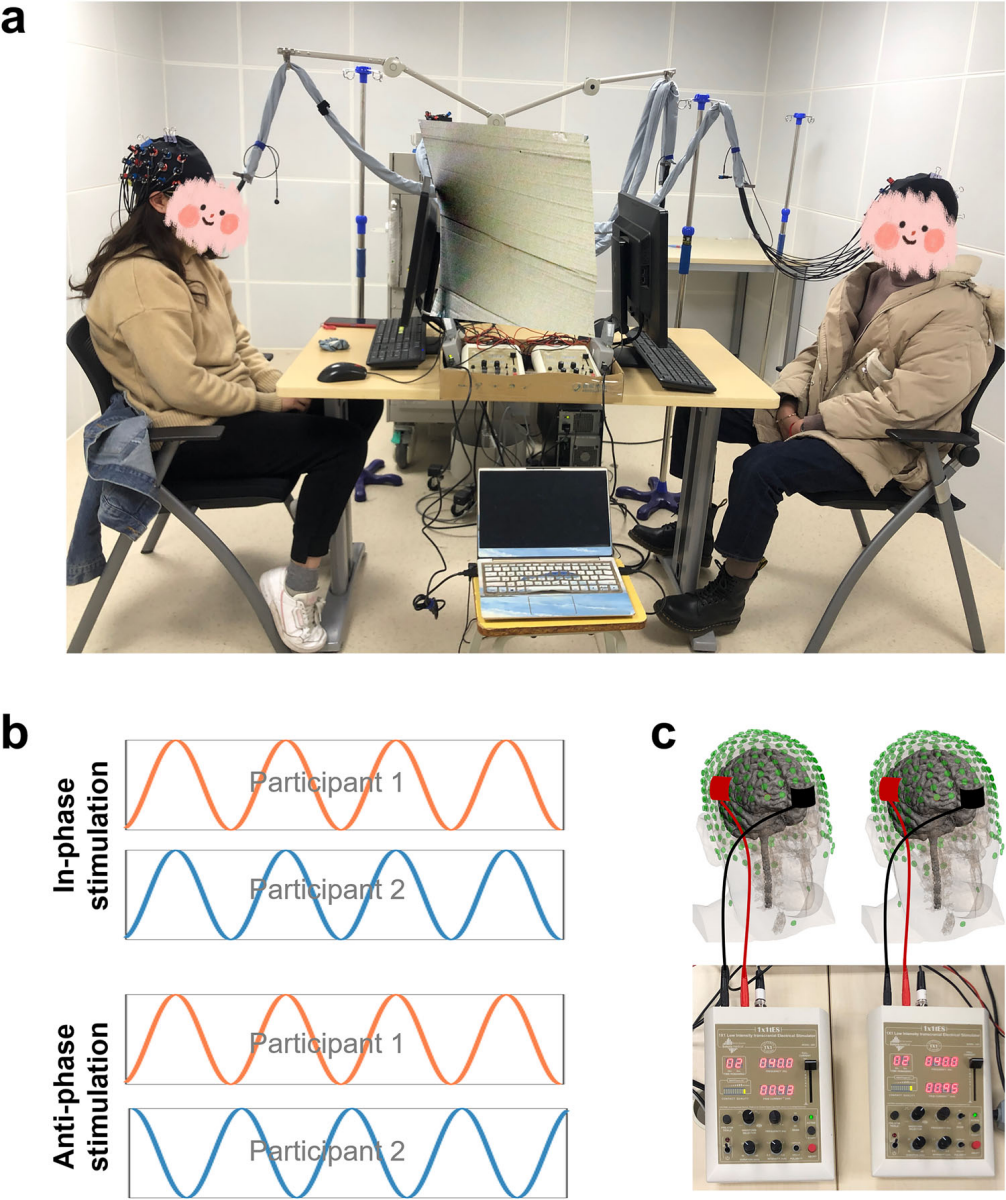
Experiment 3 design. **a** Experimental setup of the brain stimulation montage. Electrodes were placed over CP6 (anode) and FP1 (cathode), according to the international 10/10 system. **b** The relative stimulation phase between the brains was manipulated to be either in phase (0 relative phase) or anti-phase (180 relative phase). **c** Dual brain stimulation was administered through simultaneous tACS to dyads of participants prior to the CSCG with the fNIRS recording.

Experiment 2 supports the idea that the INS at the rSTG was involved in the creation of a novel interpersonal communication system. However, this inference is tentative, based purely on correlational data. In Experiment 3, we used hyper-tACS to verify whether INS plays a causal role in the emergence of a novel communication system. Seventy dyads were recruited to perform the CSCG and simultaneously stimulated via hyper-tACS before the onset of the CSCG (Fig. 8a). The relative phase of the induced oscillations was controlled to be perfectly in-phase or anti-phase (Fig. 8b) by simultaneous tACS (Fig. 8c). Sham stimulation was also used as a control condition to exclude the placebo effect.

**Fig. 9:**
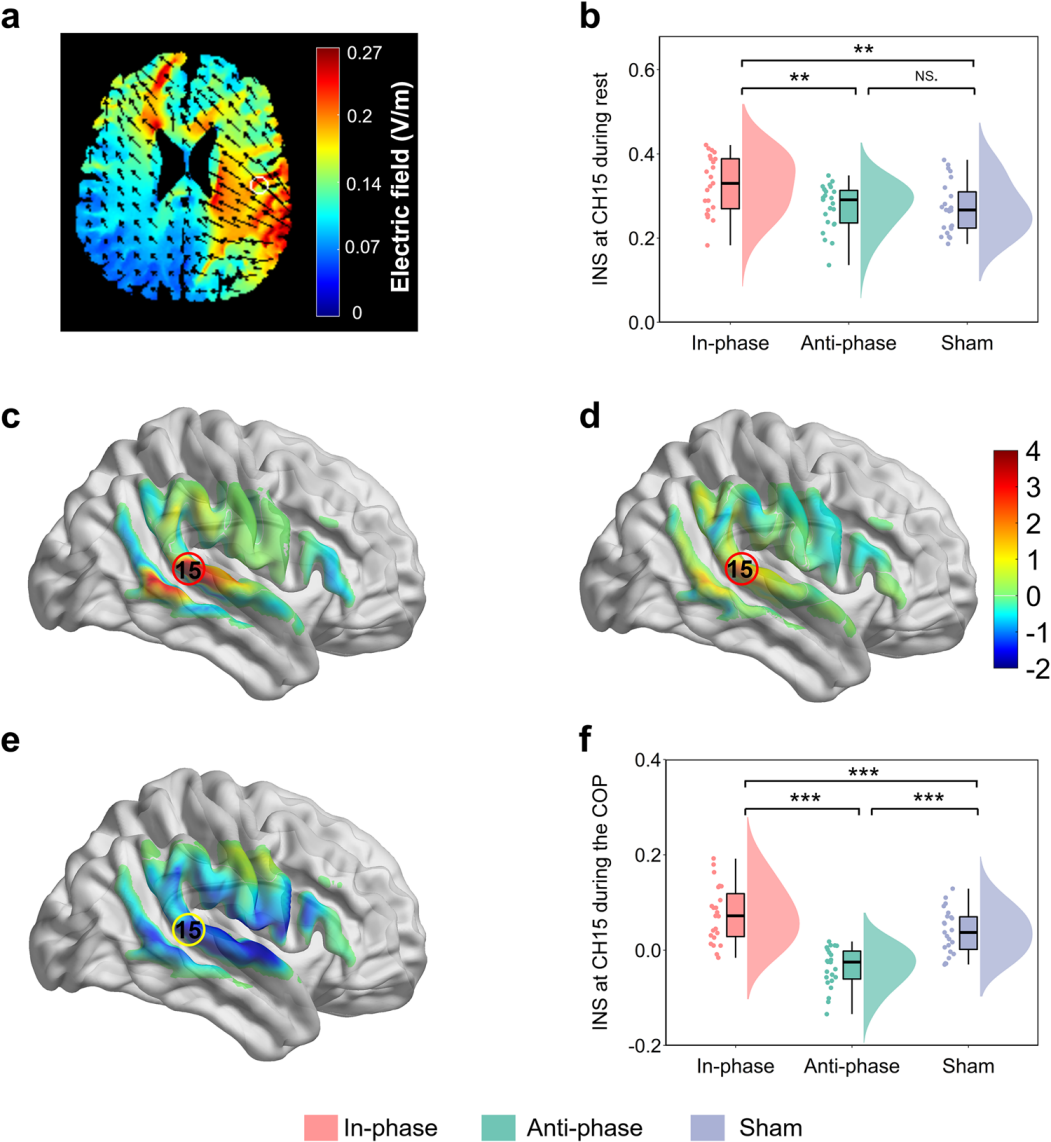
INS enhancement during the task in Experiment 3. **a** Electric field distribution on the brain. **b** Differences in INS during the resting period among the three conditions were compared by one-way analysis of variance (ANOVA). **c** One-sample *t*-test map of INS in the right temporal-parietal regions under the in-phase condition (two-tailed, FDR correction). **d** One-sample *t*-test map of INS in the right temporal-parietal regions under the sham condition (two-tailed, FDR correction). **e** One-sample *t*-test map of INS in the right temporal-parietal areas under the anti-phase condition (two-tailed, FDR correction). **f** Differences in INS during the COP among the three conditions were compared using a one-way analysis of variance (ANOVA). Data are plotted as violin and box plots for each group, with white dots indicating median values, boxes indicating 25% and 75% quartiles, and whiskers indicating the 2.5–97.5% percentile range. INS interpersonal neural synchronization, COP coordination period; *p < 0.05, **p < 0.01, ***p < 0.001 and NS not significant.

The tACS montage was determined based on computational modeling using a finite element model of the brain current flow during hyper-tACS. HD-Explore software (version 2.3, Soterix Medical, New York, NY) was used to determine and display the electrode location and current intensity. As shown in Fig. 9a, tACS stimulation produced higher current intensities in the right superior temporal gyrus region of the communicator. To verify the efficacy of the stimulus, we first analyzed the group differences of INS in the baseline period among in-phase, anti-phase, and sham conditions using a one-way ANOVA test. The main effect of the experimental stimulus condition was found to be significant, *F*(2, 68) = 6.56, *p* = 0.003, ${\eta }_{{{{{{\rm{partial}}}}}}}^{2}=0.17$. Post hoc analysis with Tukey correction showed that INS during the baseline period was higher when the two brains were stimulated in-phase (*M* ± SE: 0.33 ± 0.01), as opposed to sham (*M* ± SE: 0.27 ± 0.01) and anti-phase (*M* ± SE: 0.27 ± 0.01). However, there was no significant difference between the anti-phase stimulation and the sham condition. This result also confirmed the efficacy of the stimulus to some extent (Fig. 9b).

We then wanted to confirm whether tACS significantly affected the INS in the task period compared to the baseline period. The frequency of interest selected in Experiment 3 is 0.09 ~ 0.14 Hz (7.04 ~ 11.1 s), which is the same as in Experiment 2. Within this FOI, we calculated the task-related INS, which was defined as the INS obtained by subtracting the INS of the baseline from the COP. Then, we used the one-sample *t*-test to verify the effect of the stimulus during the task. The results revealed that the INS was significantly enhanced at CH15 during the COP under the in-phase condition (*t* = 7.84, *p* < 0.001, FDR correction; Fig. 9c) and the sham condition (*t* = 4.30, *p* = 0.006, FDR correction; Fig. 9d). Besides, we also found INS significantly decreased at CH15 during the COP under the anti-phase condition (*t* = − 3.98, *p* = 0.014, FDR correction; Fig. 9e).

Moreover, a one-way ANOVA with the task-related INS was used to compare the variability among the three conditions. The results showed significant differences across the experimental conditions (*F*(2, 68) = 41.45, *p* < 0.001, ${\eta }_{{{{{{\rm{partial}}}}}}}^{2}=0.56$; Fig. 9f), with Tukey corrected post hoc tests revealing that task-related INS was significantly higher for the in-phase condition (*M* ± SE: 0.09 ± 0.01), when compared to the sham condition (*M* ± SE: 0.04 ± 0.01) and anti-phase condition (*M* ± SE: −0.04 ± 0.01), but not between sham condition and anti-phase condition (*p* > 0.05). These results suggested that in-phase stimulation enhanced the INS in the rSTG during COP.

**Fig. 10:**
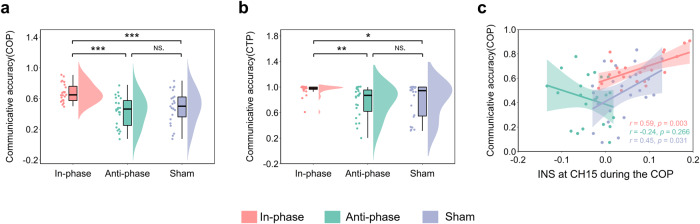
Experiment 3 behavioral results. **a** Differences in communicative accuracy during the COP among three conditions were compared using a one-way analysis of variance (ANOVA). **b** Differences in communicative accuracy during the CTP among three conditions were compared using a one-way analysis of variance (ANOVA). **c** Pearson’s correlation analyses between INS and communicative accuracy during the COP. Data are plotted as violin and box plots for each group, with white dots indicating median values, boxes indicating 25% and 75% quartiles, and whiskers indicating the 2.5–97.5% percentile range. COP coordination period, CTP communication testing period. *p < 0.05, **p < 0.01, ***p < 0.001 and NS not significant.

Our primary interest in Experiment 3 was to explore how Hyper-tACS stimulation influenced people’s communicative performance. Similarly, we conducted a one-way ANOVA with communicative accuracy during the COP and found significant differences across the experimental conditions (*F*(2, 68) = 13.12, *p* < 0.001, ${\eta }_{{{{{{\rm{partial}}}}}}}^{2}=0.29$), with Tukey corrected post hoc tests revealing that communicative accuracy was significantly higher for the in-phase condition (*M* ± SE: 0.68 ± 0.04), when compared to the sham condition (*M* ± SE: 0.43 ± 0.04) and the anti-phase condition (*M* ± SE: 0.48 ± 0.04), but not between the sham condition and anti-phase conditions (*p* > 0.9, Fig. 10a). Additionally, we conducted the Kruskal–Wallis ANOVA with communicative accuracy during the CTP and found significant differences across the experimental conditions (*H* = 13.73, *p* = 0.001), with Tukey corrected post hoc tests revealing that communicative accuracy was significantly higher for the in-phase condition (*M* ± SE: 0.96 ± 0.05), when compared to the sham condition (*M* ± SE: 0.78 ± 0.05) and anti-phase condition (*M* ± SE: 0.77 ± 0.05), but not between the sham condition and anti-phase condition (Fig. 10b).

Finally, we employed Pearson correlation analysis to examine the correlation between INS at CH15 and communication accuracy during the COP. The results showed that there was a significant positive correlation between INS at CH15 and communicative accuracy during the COP in the in-phase condition (*r* = 0.59, *p* = 0.003) and the sham condition (*r* = 0.45, *p* = 0.031), but not in the anti-phase condition (*r* = −0.24, *p* = 0.266, see Fig. 10c).

## Discussion

This study examined the psychological and neural processes that support humans in creating a novel symbolic communication system through three experiments. In Experiment 2, we manipulated dyads’ levels of shared intentionality by asking participants to create a novel symbolic communication system (experimental condition) and to communicate with each other using a pre-established communication system (control condition). We also manipulated dyads’ levels of INS through in-phase, anti-phase, and sham stimulation. Our behavioral and neuroimaging results showed that higher communicative accuracy was associated with higher levels of shared intentionality and stronger INS in the rSTG. Furthermore, INS in the rSTG increased significantly in the success group compared to the failure group under the experimental condition, but not under the control condition. We found positive correlations between INS, shared intentionality, and communicative accuracy. Interestingly, such INS enhancement partially mediated the relationship between shared intentionality and communicative accuracy. Additionally, time series analyses showed that INS in the rSTG could differentiate the success and failure groups and predicted the trial-cumulative accuracy at the early stage of communication. Finally, we used hyper-tACS stimulation to examine the causal role of the INS enhancement in the rSTG in producing higher communicative accuracy. Taken together, these results suggest that shared intentionality can modulate the emergence of a novel symbolic communication system from scratch through enhanced INS. These findings are discussed below.

Our findings extend the “Shared Intentionality Hypothesis,” which posits that human cooperative communication rests crucially on shared intentionality^24^. Previous studies have demonstrated that shared intentionality was a key feature of human joint action, including cooperation^29^, mutual support, and mutual responsiveness^30^. However, no study has been designed to directly investigate the effect of shared intentionality on the emergence of interpersonal symbolic communication systems. Using a coordinating symbolic communication game, this study demonstrated that shared intentionality between two communicators is critical for the emergence of a novel symbolic communication system. This was shown by our stepwise regression results, in which shared intentionality was the only significant predictor of communicative accuracy during the COP, explaining 43.5% of the variance in communicative accuracy during the COP in Experiment 1. Moreover, the behavioral results of Experiment 2 also showed that higher communicative accuracy was significantly correlated with higher shared intentionality scores in the success group compared to the failure group under the experimental condition.

With the development of the “second-person” neuroscience approach, INS has been recognized as a potential neural mechanism in the context of real-time social interaction^32^ as well as interpersonal communication^22,33^. Thus, we want to explore how INS affects the emergence of a novel symbolic communication system and its relationship to shared intentionality. In this study, INS enhancement in the rSTG was found only under the experimental condition, when dyads created a novel symbolic communication system, but not in the control condition when communicators already knew the figure-character mappings. This finding is partially consistent with that of Stolk et al.^21^. They observed a stronger cross-correlation between rSTG activities during the novel than known interactions in real pairs. Our control condition was similar to these “known” interactions. Still, it was aimed to rule out the potential confounding effects of the same visual and motor inputs on INS enhancement^21^. Our data extended these previous findings by showing that INS could successfully discriminate the success group from the failure group when dyads created a novel symbolic communication system from scratch.

Given the well-established link between shared intentionality, INS enhancement, and cooperative outcomes^30,34,35^, we hypothesized that dyads with higher shared intentionality would be more likely to evoke stronger INS, which was necessary for communication success. As expected, this study confirmed that INS enhancement in the rSTG was significantly correlated with communicative accuracy and shared intentionality in the success group as opposed to the failure group under the experimental condition. Our study provided complementary evidence for INS enhancement during social interactions, such as verbal communication^36^, teacher-student interaction^37^ as well as cooperation^35^, which identified a critical role for INS in the emergence of a novel symbolic communication system. Furthermore, we also observed a positive correlation between INS and shared intentionality. This result was consistent with the existing studies, which suggested that shared intentionality could increase the INS in problem-solving with shared intentionality^30^ and coordination tasks^38^. Most importantly, we found that INS was a mediator of the relationship between shared intentionality and communicative accuracy in the success group under the experimental condition.

Notably, in this study, INS enhancement was found in the rSTG, which largely overlaps with the right temporoparietal junction (rTPJ). The rTPJ is considered a key hub in the ToM processes^39^, such as inferring about the intentions and thoughts of others. Our findings confirmed previous studies that INS arising from the rSTG were found to contribute to mutual understanding during communicative interactions^21,33^. Our results are consistent with previous findings that during verbal communication, INS occurs in both low-order brain areas (A1+) and high-order brain areas (STG/STS and MTG)^23,40^. However, increased INS between speaker and listener was correlated with speech comprehension scores only in the STG/STS and not in the A1+^22^. Taken together, these results support our hypothesis that shared intentionality can improve INS enhancement between two communicators and consequently result in successful communication.

Interpersonal communication is a dynamic and ongoing process. We conducted time series analyses to explore how early INS could differentiate the success or failure group and correlate with communication accuracy. Our results showed that the difference in time-cumulative INS between the success group and the failure group became significant, started at the 9th trial, and persisted until the end of the COP period. Moreover, the time-cumulative INS in the success group was positively correlated with trial-cumulative communication accuracy starting in the 8th trial. These results were consistent with previous studies in teacher-student interaction^37,41^, which suggested that INS reached significance and was associated with teaching outcomes at a very early stage during the teaching task. Furthermore, our results suggest that INS enhancement in the rSTG may mark the communicative outcomes at the beginning of the communication process.

The current results have demonstrated a significant correlation between increased INS and communicative accuracy, but how to infer the causal relationship between them is worthy of in-depth investigation. Interpersonal communication is assumed to be a time-varying process during which communicators should adjust their mappings and decisions through current and previous feedback. Based on this, the LSTM network can handle the time-varying properties of interpersonal communication. Therefore, the LSTM should be an excellent choice for predicting trial-cumulative communication accuracy based on time-cumulative INS. Our results indicated that the time-cumulative INS successfully predicted the trial-cumulative communication accuracy. The correlation between the observed and predicted values was significant, and the random label permutation test also confirmed this result. This finding was in accordance with the previous studies, which revealed that INS as a neural marker was able to reliably discriminate the leader-follower pairs from the follower-follower pairs during a leaderless group discussion^42^.

Crucially, we applied the hyper-tACS stimulation to investigate the causal role of the INS enhancement in the rSTG producing higher communicative accuracy. Specifically, in-phase stimulation not only enhanced INS in the rSTG, but also improved communicative accuracy compared to the sham or anti-phase stimulation. This provides causal evidence that is partially consistent with previous studies^43,44^. We employed the tACS protocol to test whether synchronizing stimulation phase on two individuals’ motor cortices (M1) is sufficient to enhance interpersonal behavioral synchronization. It was reported that in-phase 20 Hz stimulation facilitated the establishment of synchronous movement. In addition^44^, Pan et al. targeted the inferior frontal cortex (IFC) of dyads composed of an instructor and a learner using a dual brain stimulation protocol. They found that 6 Hz in-phase stimulation improved learning performance more than sham stimulation. However, it should be noted that previous studies did not directly measure the effects of tACS on neural processing. In the current study, we also recorded the brain activity of two communicators in the dyads after the hyper-tACS protocol. To verify the effectiveness of the stimulus, we first analyzed the group differences in INS during the baseline period among the three conditions using a one-way ANOVA test. We observed that INS during the baseline period was higher when the two brains were stimulated in-phase than sham and anti-phase. Our findings provided additional evidence for previous studies by directly measuring tACS effects on INS in rSTG during the baseline and CSCG periods in the present experiment.

Several limitations of this study should be mentioned. First, although the design of our task provided important insights into the emergence of a novel symbolic communication in the laboratory, the computer-based paradigm limited communicators’ interactions to some extent. Our daily communication is rich in back-and-forth communicative exchanges via speech, gaze, gesture, and emotion^45^. Future research should consider these non-verbal interactions (i.e., eye contact and gesture) involved in the actual process of interpersonal communication. Second, due to the limited channels of fNIRS, we focused mainly on the right temporal regions, including rPFC and rSTG/rTPJ, rather than the whole of the brain regions involved in the ToM (e.g., vmPFC), as well as some left brain areas. We made this decision based on the similar experimental semiotic findings of refs. ^20,21^. Several relevant studies also have also found significant INS increases mainly in the rTPJ in different contexts of human interaction, such as verbal communication^46^, social decision-making^47^, and mutual understanding^21^. Notably, the vmPFC^48^, IFG^49^, and left temporoparietal regions^42,50^ are all involved in the understanding of others’ intentions. Future studies are encouraged to consolidate our findings using the MEG, which has both appropriate spatial and temporal resolutions. Finally, due to the limited number of participants, we were unable to compare the behavioral and brain results between the success and failure groups in Experiment 3. In future studies, we will increase the sample size and conduct a more detailed analysis of the differences between the success and failure groups under different stimulus conditions.

In summary, by combining CSCG, behavioral research, fNIRS-based hyperscanning, and hyper-tACS techniques, the present study revealed the psychological and neural processes that underlined the emergence of a novel symbolic communication system from scratch. During the emerging period (COP), shared intentionality increased the INS in the rSTG, resulting in the successful establishment of the communication system. Our findings extended the “shared intentionality hypothesis” by showing that shared intentionality modulated the emergence of a novel symbolic communication system through INS enhancement. Moreover, the time series, LSTM, and hyper-tACS results provided reliable evidence that INS can serve as a potential neural marker for predicting communication outcomes during the dynamic process.

## Methods

### Participants

A total of 232 participants were recruited for one of three different experiments. Experiment 1 tested the psychological processes that underline the emergence of a novel interpersonal communication system (*n* = 60, 38 females, age 18–25 years, *M* = 20.98, SD = 2.29). Experiment 2 was designed to investigate the neural processes (INS) that underline the emergence of a novel interpersonal communication system (*n* = 92, 58 females, age 18–30 years, *M* = 22.05, SD = 2.39; 6 participants were removed due to bad signal quality). Experiment 3 was designed to test the causal role of the INS during the emergence of a novel interpersonal communication system (*n* = 140, 99 females, age 18–30 years, *M* = 22.41, SD = 2.77; 2 participants were removed due to poor signal quality). All participants had normal or corrected-to-normal vision. None of them had any history of neurological or mental disorders. Participants were randomly paired into same-gender dyads and had not met each other before the experiment. This study was approved by and performed following the guidelines of the University Committee on Human Research Protection at East China Normal University. All participants provided written informed consent for the experiment.

Experiment 1: shared intentionality is the key psychological process for creating a novel interpersonal communication system

### Experimental materials

In the current study, we improved the previous experimental semiotics paradigm and designed a coordinating symbolic communication paradigm (CSCG). Two communicators were required to create a novel interpersonal communication system using a list of figures and characters. These figures varied in three dimensions: shapes (triangle, square, pentagon), colors (red, yellow, green), and inserts (heart, star, cross), which is similar to the article^51^ (Supplementary Fig. 4a). To each of the figures in the screen, participants must assign a character from nine two-syllable characters (Supplementary Fig. 4b). The task consists of two periods. The first period is the coordinating period (COP). The communication targets result from the combination of two dimensions (shape-color, shape-insert, or color-insert; 27 in total, Supplementary Fig. 4c). The second period is the communication testing period (CTP), which presents the combination of three dimensions (shape-color-insert, 27 in total, Supplementary Fig. 4d). The figures and characters were introduced in different order for two communicators in the thinking period and in each trial, which ensured that the spatial arrangement could not be used as a cue for figure-character associations during the task.

### Experimental procedures

Generally, the entire CSCG consisted of six periods, including an initial rest (3 min), a thinking period (1 min), a coordination period (COP, 27 trials, about 30–45 min), a middle rest (1 min), a communication testing period (CTP, 18 trials, about 10–20 min), and a final rest (30 s). During the thinking period, senders and receivers were instructed to familiarize themselves with the characters and figures used in the experiment. Two communicators were not required to memorize all characters and figures. The mapping relationships between figures and characters were presented differently to the sender and receiver.

Specifically, two communicators alternated as sender and receiver in a pseudo-randomized order. Each trial of COP unfolded as follows. At the beginning of the trial, each communicator was assigned a role. After role assignment, the sender was privately shown a target figure randomly selected from twenty-seven possible combinations of shape-color, shape-insert, and color-insert. Next, the sender selected two characters from nine characters displayed on a screen in a 3-by-3 grid (duration: unlimited time, see Supplementary Fig. 4b). Participants were told beforehand that each character could only represent only one figure and that they could not select the same character more than once. When the sender clicked the “OK” button, the sender and receiver would simultaneously see the characters on the screen. Next, the receiver decoded the received characters into figures by selecting a shape, color, or insert arranged in a 3-by-3 grid (duration: unlimited time, see Supplementary Fig. 4a). After that, the same feedback was presented to both sender and receiver. The feedback indicated whether the participants had matched figure-character mapping (green tick) or not matched mapping (red cross) in that trial separately. Finally, there was an interval between the two trials of the COP. The COP ended when a dyad had completed 27 trials.

The primary purpose of the COP was to establish a novel communication system through collaboration. However, the primary purpose of the CTP was to test whether or not the two participants in the COP had succeeded in establishing a novel communication system. Therefore, each trial of the CTP was designed similarly to the COP. The target presented to the sender consisted of three figures of different dimensions arranged in sequential order as shape-color-inserts. More importantly, there is no feedback to the sender and receiver for each trial. After the experiment, participants were instructed to write down what they thought the figure-character mappings were, depending on their interactions during the task (Supplementary Fig. 5).

Three essential features of this task should be highlighted. First, both senders and receivers knew that they had unlimited time available for the encoding and decoding stage. Second, communicators could not select the same character in the encoding stage or the exact figure in the decoding stage. Third, characters and figures were presented to the sender and receiver in a random order on each trial, thus avoiding the use of spatial arrangement as a cue for figure-character associations during the task.

### Experiment measurements

General characteristics: prior to the experiment, each participant completed demographic questionnaires including age, gender, education level, and so on.

Big Five Personality Scales: the personality factors were assessed using the NEO-Five Factor Inventory (NEO-FFI) developed by Morrison^52^, which consists of 60 statements with 12 items for each of the Big-Five factors of extraversion, agreeableness, conscientiousness, openness, and neuroticism^52^. Participants were asked to read each statement and rate it on how well they believed it described them on a five-point scale (1: very inaccurate to 5: very accurate). The alpha reliabilities for NEO-FFI range from 0.66 to 0.84.

Interpersonal Reactivity Index: the Interpersonal Reactivity Index (IRI) (Davis^53^) was used to measure multiple cognitive and affective components of empathy of participants, including perspective-taking, fantasizing, empathic concern, and personal distress^53^.

Need for Cognition scale: the shortened 18-item version of the Need for Cognition scale was used to measure participants’ enjoyment of thinking^54^.

Working memory test: the Operation span task (OSPAN) is a working memory task that requires participants to alternate between a set of arithmetic problems and letters for a later recall task^55^. Participants are presented with each equation and asked to judge whether it is true or false, then see a letter. This equation-letter sequence is repeated three to seven times for each trial. At the end of each trial, participants were asked to recall, in correct serial order, the letters that had preceded them during the trial. During the task, participants received feedback regarding each trial letter recall accuracy, each trial equation verification accuracy, and cumulative equation verification accuracy. Participants were asked to maintain a cumulative equation verification accuracy of at least 85% correct.

Shared intentionality scale: participants filled out a shared intentionality scale with five questions extracted from the rapport questionnaire^56^. For example, “When I was interacting with my partner, there was a shared flow of thoughts and feelings” (Supplementary Table 1). The items are rated on a seven-point Likert-scale from one (“not at all”) to seven (“extremely”).

### Data analysis

Behavioral data were analyzed using SPSS 26 (IBM Corp., Armonk, NY, USA). First, the mean score of a dyad was calculated by averaging the rating scores of two participants. In addition, communicative accuracy was defined as the number of matched figure-character mappings divided by the number of all mappings in the COP and CTP separately. Then, correlations among Big Five Personality, Interpersonal Reactivity Index, Need for Cognition, working memory, shared intentionality, and communicative accuracy during the COP were identified using Pearson’s correlation coefficient. Finally, we performed stepwise regression analyses to identify the factors that influence communicative accuracy. We set a regression model with the variables that were significantly associated with communicative accuracy in the correlation analysis. All statistical analyses were two-tailed; *p* values < 0.05 were considered statistically significant.

Experiment 2: INS partially mediates relationships between shared intentionality and communicative accuracy during the COP

### Stimuli and procedure

The stimuli and experimental procedure were the same as those in Experiment 1. Participants in a dyad sat across from each other at a table with two computer screens on which the stimuli were displayed. We placed a board between the two computer screens so that each could not see the other during the session.

According to the operational definition of shared intentionality, there are three dimensions: shared goals, collaborative interaction, and shared mental states. In the experimental condition, the given characters are meaningless, and there are no pre-specified correspondences between the characters and the figures. Thus, the two communicators have to interact and collaborate with each other using figures and characters and guess each other’s thoughts through feedback information. The purpose of the experimental condition was to build a novel interpersonal symbolic communication system from scratch. In the control condition, communicators were told in advance which character corresponded to each figure. Specifically, the nine English characters in the control condition became the abbreviations corresponding to the related English words that were conveniently memorized before the control condition for these nine figures: triangle-th (three), quadrangle-fo (four), pentagon-fi (five), red-re, yellow-ye, green-gr, heart-he, cross-cr, star-st. The experimenter explicitly told both communicators the correspondence between these figures and characters, and through the practice phase ensured that each communicator fully mastered the correspondences between these figures and characters. Overall, the three dimensions of shared intentionality were present in the experimental condition, and therefore the level of shared intentionality should be higher. However, the level of shared intentionality should also be lower in the control condition, in which two communicators had a common goal and had to interact collaboratively, but they did not have to share their mental states with each other. We also tested the reliability of the manipulation by measuring the level of shared intentionality in each of the two conditions using the questionnaire.

The success and failure groups are defined according to the post-experimental questionnaire. If two communicators in a dyad agree on a one-to-one correspondence for all these 9 figures and 9 characters and the communicative accuracy of the CTP reaches 80% or more, we define them as the success group. Otherwise, we define them as the failure group.

The oxygenated hemoglobin (Hbo) and deoxygenated hemoglobin (Hbr) signals were acquired using a continuous wave fNIRS system (LABNIRS, Shimadzu Corporation, Kyoto, Japan). For each participant, 15 optodes were placed in the right temporal-parietal region, a “3 × 5” probe patch forming 22 channels. The distance between the emitter optodes and detector optodes was set to 3 cm. As the reference site, the middle optode in the lowest line was located at the T4 in the international 10-20 system. The row of the probe was aligned along the sagittal reference plane. Five anatomical cranial reference positions (Nz, Cz, Iz, left, and right preauricular), 15 probes, and 22 channels in real space were acquired by employing the 3D digitizer. To identify the anatomical location of each channel in our study, Montreal Neurological Institute (MNI) coordinates were calculated for each emitter, detector, and channel position using the NIRS_SPM software^57^. Structural labels for the Brodmann area (BA) coordinates of each channel are shown in Supplementary Table 2.

### Behavioral data analysis

The behavioral data were analyzed using SPSS 26 (IBM Corp., Armonk, NY, USA), which was consistent with Experiment 1. Before analysis, we checked whether the data distribution was normal or not. If the data distribution was normal, two-tailed Student’s *t*-test and Pearson’s correlation were used. If it was not, the Wilcoxon signed rank test and Spearman’s rank correlation were used.

### fNIRS data analysis

In the present study, the fNIRS data collected during the task and at rest were analyzed based on the platform of Matlab 2020b (Mathworks Inc., Natick, MA, USA). During preprocessing, the data were processed using the Homer2 package in MATLAB. First, the motion artifacts were detected using the function hmrMotionArtifactByChannel. After that, the raw intensity data were converted to optical density (OD) changes. Then, Kurtosis-based wavelet filtering was applied to remove motion artifacts with a threshold of 3.3, as suggested in the original paper^58^. After this step, a bandpass filter (0.01 to 1 Hz) was conducted to reduce low frequency drifts and high frequency noise. Then the OD data were converted to the Hbo concentration using the modified Beer-Lambert law.

After these preprocessing steps, wavelet transforms coherence (WTC) was used to assess the correlation coefficient of two Hbo time series generated by each dyad^59^. In the current study, we chose the INS in the middle 2 min of the initial rest period as the baseline. First, we calculated the average INS of all dyads for each channel in the frequency band ranging from 0.01 to 1 Hz. Second, the INS of the baseline was subtracted from that of the task period (from the beginning to the end of each trial) and converted into *z*-scores using Fisher *z*-statistics. Third, a series of one-sample *t*-tests were conducted on all frequency bands of the channels, and the 0.10–0.12 Hz frequency with a threshold at *p* < 0.05 with FDR correction was selected. After that, the frequencies around this frequency whose *p* values were <0.05 were also considered. Finally, the final frequency of interest (FOI) ranged from 0.09 to 0.14 Hz (7.04–11.10 s).

Task-related INS was defined as the INS obtained by subtracting the baseline INS from the COP in this study. Then, the trial-averaged INS for the task period relative to baseline within the FOI was calculated and converted into *z*-scores using Fisher *z* statistics. After that, a one-sample *t*-test with FDR correction (*p* < 0.05) was then performed again on the task-related INS for each channel to identify those channels that showed significance during the task. All subsequent statistical tests were conducted on the task-related INS enhancement. To verify that the INS enhancement was not obtained by chance, we permutated the time series of two participants from each dyad. A reanalysis of the INS on the obtained randomized time series was conducted as a control analysis. It is also worth mentioning that successful communication required each dyad to share and infer the figure-character mappings through feedback, which only existed during the COP. Therefore, our subsequent analysis focused mainly on the INS and communicative accuracy during the COP.

### Mediation effect analysis

To examine the potential mediation of the INS enhancement on the association between shared intentionality and communicative accuracy, we conducted a mediation analysis^60^. This analysis assumes that the independent variable (X) affects the mediator (M), which then affects the dependent variable (Y). It is an extension of simple linear regression in that one or more variables are added to the regression equation. In the current study, the simple mediation effect was tested by PROCESS model 4 using the bootstrapping method with 5000 bootstraps resamples^61^. In the analysis, shared intentionality and communicative accuracy were entered as input and output variables, respectively, and the INS was entered as the mediating variable. A series of linear regressions were used to assess (1) the effect of shared intentionality on INS (represented by *a*); (2) the effect of INS on communicative accuracy after removing the effect of shared intentionality (represented by *b*); and (3) the total effect of shared intentionality on communicative accuracy (represented by *c*). The indirect effect, that is *ab*, was statistically significant if the confidence interval did not include zero.

### Dynamics of the INS analysis and neural-behavioral correlation in timeseries

We conducted time-series analyses to identify the earliest trial whose INS enhancement correlated with communicative accuracy. First, a time-cumulative INS during the COP was calculated for these channels. The time-cumulative INS at trial *n* was calculated as the average of the INS ranging from the first trial to the *n*th trial. Second, the trial-cumulative accuracy was calculated by dividing the number of matched mappings by the number of all mappings from the start to the end of the *n*th trial. Third, two-sample *t*-tests were conducted on the time-cumulative INS enhancement and trial-cumulative accuracy between the success and failure groups, respectively. Fourth, correlation analyses between time-cumulative INS and trial-cumulative accuracy were performed for each trial. For the analyses of the third and fourth steps, the resulting *p* values were corrected by FDR. The same analyses were also conducted for the control condition and the randomized time series Hbo data.

### Prediction of communicative accuracy

The above analysis was used to explore whether the INS correlates with communicative accuracy. However, the causal relationship between them requires further analysis. To explore the possibility that the INS enhancement could significantly predict communication accuracy, we conducted a long short-term memory (LSTM) neural network in this study. LSTM is a special type of Recurrent Neural Network (RNN) proposed by Hochreiter and Schmidhuber in 1997^62^. It has some gated units (i.e., input gate, forget gate, and output gate) that further improve the ability to capture the long-term dependencies of the data.

Each LSTM unit contains a cell state *c*_*t*_ and hidden state *h*_*t*_. The first step is to select what to throw away from the input *x*_*t*_ and the previous hidden state *h*_*t*−1_, which is made by the forget gate *f*_*t*_. The second step is performed by the input gate by two steps, consisting of a sigmoid layer and a tanh layer. It determines what information will be stored in the cell state. The third step is to update the cell state. That is *C*_*t*−1_ is updated to *C*_*t*_. Finally, the output gate decides what is the output information *h*_*t*_ for the new state by *o*_*t*_. The process is based on four fully connected neurons, and the equations governing each LSTM cell are shown below:

1 $${f}_{t}=\sigma \left({W}_{f}\cdot \left[{h}_{t-1},{x}_{t}\right]+{b}_{f}\right)$$

2 $${i}_{t}=\sigma \left({W}_{i}\cdot \left[{h}_{t-1},{x}_{t}\right]+{b}_{i}\right)$$

3 $${\tilde{C}}_{t}=\tanh \left({W}_{C}\cdot \left[{h}_{t-1},{x}_{t}\right]+{b}_{C}\right)$$

4 $${C}_{t}={f}_{t}* {C}_{t-1}+{i}_{t}* {\tilde{C}}_{t}$$

5 $${o}_{t}=\sigma \left({W}_{o}\left[{h}_{t-1},{x}_{t}\right]+{b}_{o}\right)$$

6 $${h}_{t}={o}_{t}* \tanh \left({C}_{t}\right)$$

Here, we represent the corresponding weights of each input *x*_*t*_ and each previous state *h*_*t*−1_. *b* is the corresponding biases. The “*” denotes element-wise multiplication (Hadamard product). The “+” denotes the element-wise addition.

Supplementary Fig. 6 displays the overall prediction schematic flow and the detailed structure of the LSTM unit. The input is the time-cumulative INS, and the output is the trial-cumulative accuracy. First, the same preprocessing and WTC analysis procedures were applied (see fNIRS data analysis section). Second, the dyads were randomly divided into two sub-datasets of 90% (training dataset) and 10% (testing dataset). Third, the LSTM neural network was trained using the training dataset, yielding a prediction model. Fourth, this model was applied to the testing dataset to predict trial-cumulative accuracy. Last, Spearman’s rank correlation coefficient between the predicted and true values is calculated to quantify the prediction accuracy^63^.

Experiment 3: Manipulating of INS by transcranial alternating current stimulation

### Stimuli and procedure

These were the same as those in Experiment 2 except for the following. Prior to the formal experiment, participants underwent the hyper-tACS protocol. Hyper-tACS was delivered via two battery-driven stimulators (Model: 2001; Soterix Medical Inc., New York, USA). Electrode placement was based on Experiment 2 and was determined using the EEG 10-20 International System. Specifically, for the stimulation of the right STG, the center of the anode was centered on Cp6, while the cathode was positioned on Fp1^64^. A constant current of 1 mA intensity was delivered with stimulation electrodes (5 × 5 cm) in saline-soaked sponge envelopes (5 × 7 cm). The frequency was 40 Hz (i.e., gamma band) based on our previous study.

The two stimulators were controlled through Data Acquisition Toolbox Support Package for National Instruments NI-DAQmx Devices in MATLAB (MathWorks Inc., Natick, MA) via two USB/Parallel 24-Bit Digital I/O Interfaces (Model: SD-MSTCPUA; Cortech Solutions Inc., North Carolina, USA). The external trigger was sent simultaneously from the computer to the Digital I/O Interfaces to synchronize the two stimulators.

Participants were randomly assigned to one out of three experimental conditions (23 dyads for each group): (1) in-phase condition, both subjects received stimulation with a zero phase difference; (2) anti-phase condition, both subjects received stimulation with a 180-degree phase difference; and (3) sham condition, both subjects received 30 s fade-in and 30 s fade-out of stimulation. In the two real stimulation conditions, tACS was applied for 20 min. We conducted a double-blind study. Therefore both experimenters and participants were blinded by the experimental conditions. After the hyper-tACS protocol, two participants were required to complete the CSCG procedure that was the same as those in Experiment 2.

### fNIRS data acquisition and analysis

After the hyper-tACS protocol, two participants finished the CSCG. Changes in oxygenated hemoglobin (Hbo) and deoxygenated hemoglobin (Hbr) concentrations were measured, during the CSCG, using a NIRS system (ETG-7100, Hitachi Medical Corporation, Tokyo, Japan). fNIRS data acquisition and analysis were the same as those in Experiment 2.

### Statistical analysis

We conducted a one-way ANOVA of the effects of stimulation (in-phase, anti-phase, and sham) on baseline INS, task-related INS, and communicative accuracy, respectively. Correlations between shared intentionality and communicative accuracy during the COP were identified using Pearson’s correlation coefficient.

### Statistics and reproducibility

Behavioral data and INS were analyzed using SPSS 26 (IBM Corp., Armonk, NY, USA). Two-tailed Student’s *t* test (in Experiment 2) and one-way ANOVA (in Experiment 3) were used to determine the significance of the difference between the different conditions and groups. *p* < 0.05 were considered statistically significant. Correlations between behavioral indices INS and communicative accuracy during the COP were determined using Pearson’s correlation or Spearman’s rank correlation. Details of behavioral and fNIRS data analysis are provided in the “Data analysis” section.

### Reporting summary

Further information on research design is available in the Nature Portfolio Reporting Summary linked to this article.

### Supplementary information

Supplementary information

Reporting Summary

## Data Availability

The main data used to generate all the figures and analyses in this article are available on GitHub: (https://github.com/Joan8912/shared-intentionality-INS-interpersonal-communication-data). Individual behavioral and fNIRS raw data are available from the corresponding author upon reasonable request.

## Code availability

In the present study, the fNIRS data collected during the task and at rest were analyzed using the Matlab 2020b platform (Mathworks Inc., Natick, MA, USA). All custom scripts used in this study were written in Matlab 2020b and are available upon request.
