# Supplementary material for: Shared intentionality modulates interpersonal neural synchronization at the establishment of communication system
Source: Commun Biol. 2023 Aug 10;6:832. doi: 10.1038/s42003-023-05197-z (PMC10415255; doi:10.1038/s42003-023-05197-z)
Supplement: Supplementary file 1 — Supplementary information [file 42003_2023_5197_MOESM1_ESM.pdf]

Shared intentionality modulates interpersonal neural synchronization at the establishment of communication system

Liu Jieqiong[1,2], Zhang Ruqian[1], Xie Enhui[1], Lin Yixuan[1], Chen Danni[1], Liu Yang[1], Li Keshuang[1], Chen Mei[1], Li Yangzhuo[1], Wang Guanghai[2*] and Li Xianchun[1*]

[1*]Shanghai Key Laboratory of Mental Health and Psychological Crisis Intervention, Affiliated Mental Health Center (ECNU), School of Psychology and Cognitive Science, East China Normal University, Shanghai,China.

[2]Paediatric Translational Medicine Institute, Department of Developmental and Behavioral Pediatrics, Shanghai Children's Medical Center, School of Medicine, Shanghai Jiao Tong University, Shanghai,China.

*Corresponding author(s). E-mail(s): wang-guanghai@163.com; xcli@psy.ecnu.edu.cn;

1 Supplementary Information

Supplementary Table 1. Questionnaires for shared intentionality.

Items	Score		
1) When I was interacting with my partner, there was a shared flow of thoughts and feelings.	Not at all	1 2 3 4 5 6 7	Extremely
2) To what extent do you think you and the partner were aware of each other?	Not at all	1 2 3 4 5 6 7	Extremely
3) To what extent do you think you and the partner felt coordinated with each other?	Not at all	1 2 3 4 5 6 7	Extremely
4) To what extent do you think you and the partner understood each other?	Not at all	1 2 3 4 5 6 7	Extremely
5) To what extent do you think you and the partner had a feeling of mutual agreement?	Not at all	1 2 3 4 5 6 7	Extremely

Supplementary Table 2. MNI coordinates and corresponding maximum probability Brodman Talairach areas for each channel used in this study.

Channels	MNI coordinates (mm)			Brodman Talairach areas
	x	y	z	
1	56.65	-52.57	47.99	Supramarginal gyrus part of Wernicke's area
2	63.69	-28.71	46.73	Primary Somatosensory Cortex
3	59.54	-0.55	42.37	Pre-Motor and Supplementary Motor Cortex
4	49.58	27.54	37.77	Dorsolateral prefrontal cortex
5	54.26	-65.79	35.85	Angular gyrus, part of Wernicke's area
6	65.25	-41.34	36.07	Supramarginal gyrus part of Wernicke's area
7	66.97	-15.41	32.83	Pre-Motor and Supplementary Motor Cortex
8	60.89	12.90	28.12	Dorsolateral prefrontal cortex
9	50.07	41.09	22.86	Dorsolateral prefrontal cortex
10	63.26	-54.64	21.94	Superior Temporal Gyrus
11	69.11	-29.43	21.09	Supramarginal gyrus part of Wernicke's area
12	66.55	-2.33	18.42	Pre-Motor and Supplementary Motor Cortex
13	58.64	26.97	13.31	pars triangularis Broca's area
14	57.92	-68.44	7.02	Fusiform gyrus
15	69.56	-43.51	6.16	Superior Temporal Gyrus
16	70.65	-17.25	3.67	Superior Temporal Gyrus
17	61.82	7.54	1.23	Superior Temporal Gyrus
18	54.67	39.87	-3.82	Inferior prefrontal gyrus
19	64.26	-56.31	-7.46	Fusiform gyrus
20	71.27	-30.65	-9.20	Middle Temporal gyrus
21	67.60	-5.90	-13.50	Middle Temporal gyrus
22	55.67	17.84	-16.92	Temporopolar area

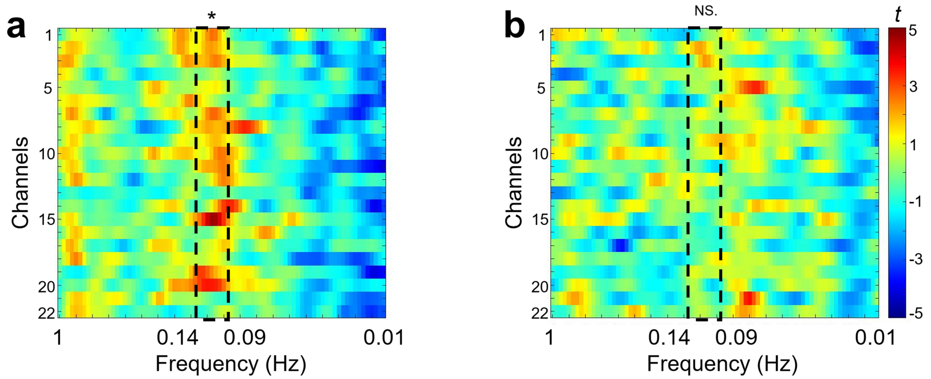

Supplementary Fig. 1. Frequency band of interest under the experimental and control condition. Interpersonal neural synchronization (INS) was calculated by wavelet transform coherence (WTC). (a) One-sample t-test map of the INS during the COP for each channel across frequencies ranging from 0.01Hz to 1 Hz, under the experimental condition (two-tailed). The black border with an asterisk represents frequency whose p values are < 0.05 (0.09 to 0.14 Hz, 7.1s–11.1s). (b) One-sample t-test map of the INS during the COP for each channel across frequencies ranging from 0.01Hz to 1 Hz, under the control condition (two-tailed). The black border with "NS." represents p values are > 0.05 in this frequency (0.09 to 0.14 Hz, 7.1s–11.1s).

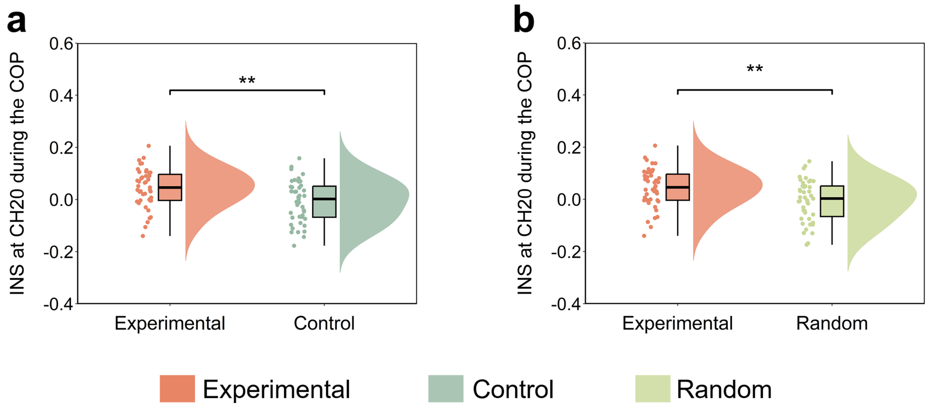

Supplementary Fig. 2. INS enhancement during task. (a) The paired samples t-test INS at CH20 under different conditions (experimental vs. control). (b) The paired samples t-test INS at CH20 under different conditions (experimental vs. random). Data are plotted as violin and box plots for each group, with white dots indicating median values, boxes indicating 25% and 75% quartiles and whiskers indicating the 2.5–97.5% percentile range. INS: interpersonal neural synchronization; COP: coordination period. $^{**}p < 0.01$.

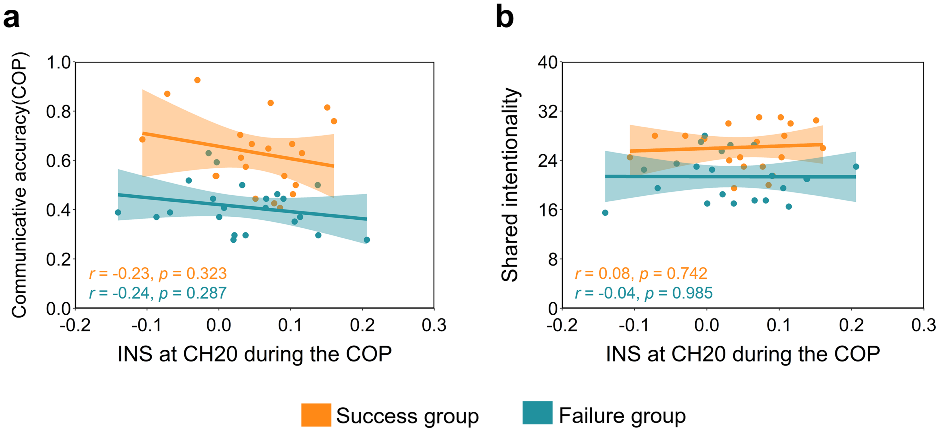

Supplementary Fig. 3. Correlations between behavioral indicators. (a) Pearson's correlation between INS at CH20 and communicative accuracy during the COP under the experimental condition in different groups (success vs. failure) under the experimental condition. (b) Pearson's correlation between INS at CH20 and shared intentionality in different groups (success vs. failure) under the experimental condition. INS: interpersonal neural synchronization; COP: coordination period.

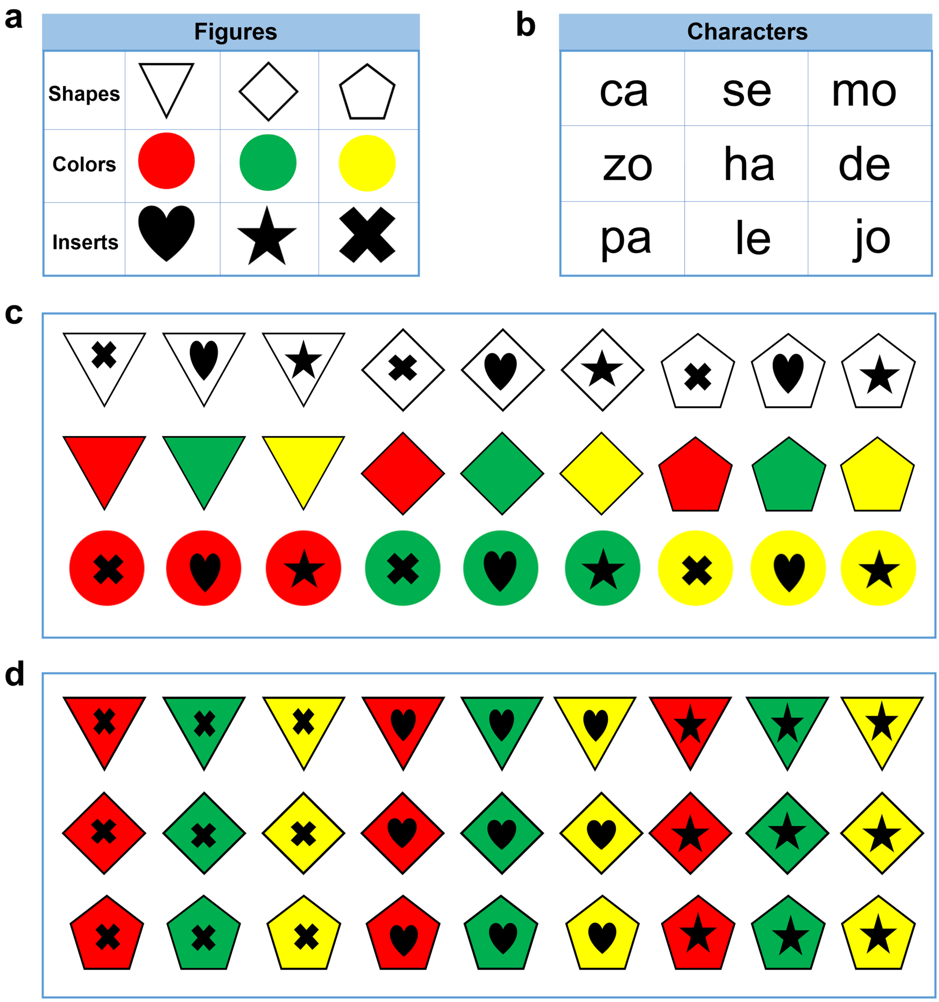

Supplementary Fig. 4. Stimuli are used in the CSCG. (a) Figures used in Experimental 1. (b) Characters used in Experimental 1. (c) Twenty-seven possible combinations of shape-color, shape-insert, and color-insert were presented in the COP. (D) Twenty-seven possible combinations of three features (shape-color-insert) were presented in the CTP. COP: coordination period; CTP: communication testing period.

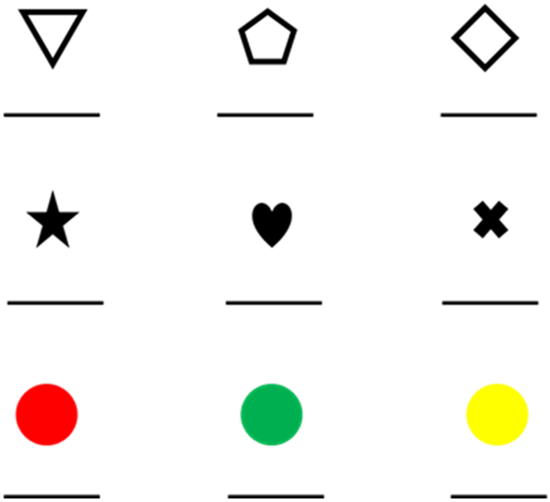

Supplementary Fig. 5. Post-experiment measurements about the figure-character mappings. Participants were given the following instruction: “Please fill in the blanks with the characters (ca, jo, ha, mo, de, pa, se, zo, le) that you used to encode the figures based on your communication during the task.”.

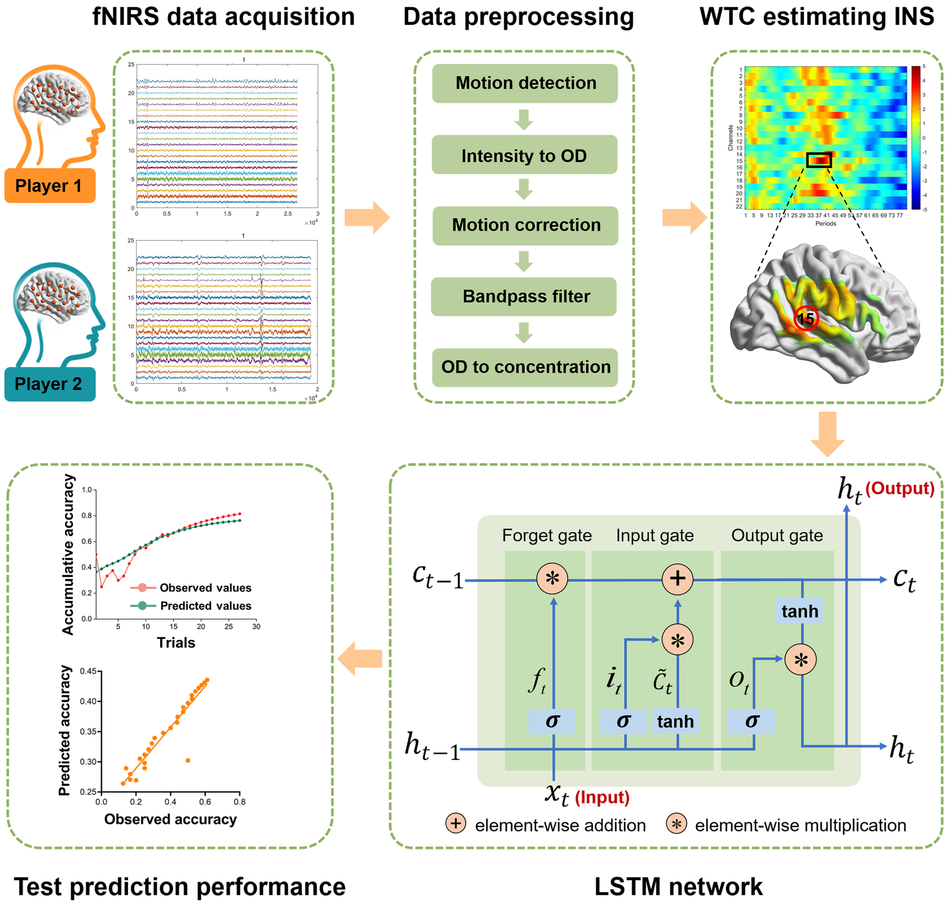

Supplementary Fig. 6. Schematic illustration of the fNIRS data pre-processing and processing, and overview of the LSTM neural network.
