# Supplementary material for: Shared intentionality modulates interpersonal neural synchronization at the establishment of communication system
Source: Commun Biol. 2023 Aug 10;6:832. doi: 10.1038/s42003-023-05197-z (PMC10415255; doi:10.1038/s42003-023-05197-z)
Supplement: Supplementary file 2 — Reporting Summary [file 42003_2023_5197_MOESM2_ESM.pdf]

nature portfolio

Corresponding author(s): Guanghai Wang and Xianchun Li

Last updated by author(s): Jul 23, 2023

Reporting Summary

Nature Portfolio wishes to improve the reproducibility of the work that we publish. This form provides structure for consistency and transparency in reporting. For further information on Nature Portfolio policies, see our Editorial Policies and the Editorial Policy Checklist.

Statistics

For all statistical analyses, confirm that the following items are present in the figure legend, table legend, main text, or Methods section.

n/a	Confirmed	
☐	☒	The exact sample size (*n*) for each experimental group/condition, given as a discrete number and unit of measurement
☐	☒	A statement on whether measurements were taken from distinct samples or whether the same sample was measured repeatedly
☐	☒	The statistical test(s) used AND whether they are one- or two-sided<br>*Only common tests should be described solely by name; describe more complex techniques in the Methods section.*
☒	☐	A description of all covariates tested
☐	☒	A description of any assumptions or corrections, such as tests of normality and adjustment for multiple comparisons
☐	☒	A full description of the statistical parameters including central tendency (e.g. means) or other basic estimates (e.g. regression coefficient) AND variation (e.g. standard deviation) or associated estimates of uncertainty (e.g. confidence intervals)
☐	☒	For null hypothesis testing, the test statistic (e.g. *F*, *t*, *r*) with confidence intervals, effect sizes, degrees of freedom and *P* value noted<br>*Give P values as exact values whenever suitable.*
☒	☐	For Bayesian analysis, information on the choice of priors and Markov chain Monte Carlo settings
☒	☐	For hierarchical and complex designs, identification of the appropriate level for tests and full reporting of outcomes
☐	☒	Estimates of effect sizes (e.g. Cohen's *d*, Pearson's *r*), indicating how they were calculated

Our web collection on statistics for biologists contains articles on many of the points above.

Software and code

Policy information about availability of computer code

Data collection	The behavioral data collection used the E-prime 2.0 (version 2.0.10.353, Psychology Software Tools Inc, Pittsburgh, PA,USA).<br>The changes in oxygenated hemoglobin (Hbo) and deoxygenated hemoglobin (Hbr) concentrations were measured, during teaching tasks, using a continuous wave fNIRS system (LABNIRS, Shimadzu Corporation, Kyoto, Japan) and a NIRS system (ETG-7100, Hitachi Medical Corporation, Tokyo, Japan).
Data analysis	In the present study, the fNIRS data collected during the task and at rest were analyzed based on the platform of Matlab 2020b (Mathworks Inc., Natick, MA, USA). The behavioral data and INS were analyzed using SPSS 26 (IBM Corp., Armonk, NY, USA).

For manuscripts utilizing custom algorithms or software that are central to the research but not yet described in published literature, software must be made available to editors and reviewers. We strongly encourage code deposition in a community repository (e.g. GitHub). See the Nature Portfolio guidelines for submitting code & software for further information.

Data

Policy information about availability of data

All manuscripts must include a data availability statement. This statement should provide the following information, where applicable:

- Accession codes, unique identifiers, or web links for publicly available datasets
- A description of any restrictions on data availability
- For clinical datasets or third party data, please ensure that the statement adheres to our policy

The data and analysis code in this study are available from the corresponding author upon reasonable request.

Research involving human participants, their data, or biological material

Policy information about studies with human participants or human data. See also policy information about sex, gender (identity/presentation), and sexual orientation and race, ethnicity and racism.

Reporting on sex and gender	A total of 232 university students were recruited for one of three different experiments. Experiment 1: n = 60, 38 females, age 18 to 25 years; Experiment 2: n = 92, 58 females, age 18 to 30 years; Experiment 3: n = 140, 99 females, age 18 to 30 years. In this study, we collected gender information from participants via a self-report questionnaire before the start of each experiment. Participants were grouped to ensure that participants assigned to the same group were of the same sex. No sex/gender analyses were performed.
Reporting on race, ethnicity, or other socially relevant groupings	The participants' personal information was collected through a self-report questionnaire before the experiment began, and the relevant terms used in the experiment were provided by the experimenter. In this study, all the subjects were undergraduate students of East China Normal University. All subjects were Chinese, belonging to the Asian group.
Population characteristics	See above
Recruitment	The participant recruitment advertisement was posted on various social networking sites and dedicated participant recruitment platforms to inform potential participants about the recruitment information. In addition, the researcher specified the criteria for selecting potential participants in the recruitment information and confirmed whether potential participants met the recruitment criteria when they contacted the researcher.
Ethics oversight	This study was approved by and performed following the guidelines of the University Committee on Human Research Protection at East China Normal University.

Note that full information on the approval of the study protocol must also be provided in the manuscript.

Field-specific reporting

Please select the one below that is the best fit for your research. If you are not sure, read the appropriate sections before making your selection.

☐ Life sciences ☒ Behavioural & social sciences ☐ Ecological, evolutionary & environmental sciences

For a reference copy of the document with all sections, see nature.com/documents/nr-reporting-summary-flat.pdf

Behavioural & social sciences study design

All studies must disclose on these points even when the disclosure is negative.

Study description	Quantitative experimental study
Research sample	A total of 232 university students were recruited for one of three different experiments. Experiment 1 tested the psychological processes that underline the emergence of a novel interpersonal communication system (n = 60, 38 females, age 18 to 25 years, M = 20.98, SD = 2.29). Experiment 2 was designed to investigate the neural processes (INS) that underline the emergence of a novel interpersonal communication system (n = 92, 58 females, age 18 to 30 years, M = 22.05, SD = 2.39). Experiment 3 was designed to test the causal role of the INS during the emergence of a novel interpersonal communication system (n = 140, 58 females, age 18 to 30 years, M = 22.41, SD = 2.77). All participants had a normal or corrected-to-normal vision. None of them had any history of neurological or mental disorders.
Sampling strategy	In Experiment 2, we first estimated the sample size by setting the effect size (Cohen's d) for the paired samples t-test to 0.5, with a target power of 0.8 and an alpha level of 0.05. The total sample size was 34. In addition, we also estimated the sample size by setting the effect size (Cohen's d) derived from a preliminary experiment to 0.9, with a target power of 0.8 and an alpha level of 0.05 for the independent samples t-test. The required sample size was 21 for each group, which in our study was 21 and 22, respectively.<br>Since no relevant studies based on hyper-tACS technique and one-way ANOVA were found. In Experiment 3, we focused more on whether in-phase stimulation significantly enhanced communicative accuracy and INS compared to sham or anti-phase stimulation. We estimated the sample size by setting the effect size (Cohen's d) derived from a previous research to 0.91 (Novembre et al., 2017),

	with a target power of 0.8 and an alpha level of 0.05 for the independent samples t-test. The required sample size was 20 for each group, and we rounded up to 23 for each group.
Data collection	In Experiment 1, participants' behavioral data were collected using a pre- and post-experimental questionnaire. Participants were asked to complete the questionnaire using a pen. Behavioral data during the formal experiments were collected by computer, and the experimental procedures were presented on the computer screen using E-prime 2.0 software (version 2.0.10.353, Psychology Software Tools Inc, Pittsburgh, PA, USA). In Experiments 2 and 3, behavioral data were collected as in Experiment 1, and changes in oxygenated hemoglobin (Hbo) and deoxygenated hemoglobin (Hbr) concentrations were measured using the functional near-infrared spectroscopy (fNIRS) system.
Timing	Experiment 1 and Experiment 2 were conducted from May 2018 to March 2019. Experiment 3 ran from January 2021 to March 2021.
Data exclusions	No participants were excluded from Experiment 1. In Experiment 2, six participants were excluded due to poor fNIRS signal quality. In Experiment 3, two participants were excluded due to poor fNIRS signal quality. The exclusion criteria were based on the coefficient of variation (CV) calculated on the raw data as in the previous study.
Non-participation	No participants dropped out in these three studies.
Randomization	In Experiment 2, the success and failure groups are defined according to the post-experimental questionnaire. If two communicators in a dyad agree on a one-to-one correspondence for all these 9 figures and 9 characters and the communicative accuracy of the CTP reaches 80% or more, we define them as the success group, otherwise we define them as the failure group. In Experiment 3, participants were randomly paired into same-gender dyads and were randomly assigned to different stimulus conditions.

Reporting for specific materials, systems and methods

We require information from authors about some types of materials, experimental systems and methods used in many studies. Here, indicate whether each material, system or method listed is relevant to your study. If you are not sure if a list item applies to your research, read the appropriate section before selecting a response.

Materials & experimental systems

n/a	Involved in the study
☒	☐ Antibodies
☒	☐ Eukaryotic cell lines
☒	☐ Palaeontology and archaeology
☒	☐ Animals and other organisms
☒	☐ Clinical data
☒	☐ Dual use research of concern
☒	☐ Plants

Methods

n/a	Involved in the study
☒	☐ ChIP-seq
☒	☐ Flow cytometry
☒	☐ MRI-based neuroimaging
